# Testing before learning: Exploring the robustness of the pretesting effect

**DOI:** 10.3758/s13421-025-01813-x

**Published:** 2025-11-06

**Authors:** Yeray Mera, Nataliya Dianova, Eugenia Marin-Garcia

**Affiliations:** Department of Basic Psychological Processes and their Development, Faculty of Psychology, University of the Basque Country UPV/EHU, Tolosa Hiribidea, 70, Donostia-San Sebastián, 20018 Spain

**Keywords:** Retrieval practice, Testing effect, Pretesting, Encoding, Error learning

## Abstract

Retrieval practice, or taking tests after studying, is a highly effective strategy to enhance learning. Furthermore, pretesting, which involves attempting and failing to guess unknown information before studying it, has emerged as a more effective strategy than mere re-studying. These methods exemplify errorful learning and represent powerful learning tools. In a series of experiments, we investigated how test order – whether administered before (pretest) or after (post-test) exposure to the learning material – affects memory compared to errorless copying (Experiment 1). Experiment 2 followed a similar design but included a copy-test-copy condition to further explore the potential impacts of test order. The results revealed that the pretest, post-test, and copy-test-copy groups all improved memory compared to errorless copying, with no difference between the three. Error type analysis indicated minimal intrusion errors. In Experiment 3, we explored the pretesting effect in older adults. The results showed that pretesting significantly enhanced memory compared to copying, indicating the robustness of pretesting in healthy aging and across age groups. Interestingly, participants across the experiments consistently underestimated the efficacy of testing, revealing a gap in metacognitive awareness. These findings underscore the efficacy of errorful learning as a robust strategy with broad applicability across diverse populations and procedures.

## Introduction

In human cognition, making errors and managing failure are intrinsic components of the natural learning process. Despite this, error commission has been traditionally avoided in educational settings (Stevenson & Stigler, 1994; Tulis, 2013). Advocates of errorless learning have emphasized the need to minimize errors during learning to prevent their reinforcement in memory (Bandura, 1986; Skinner, 1953, 1958). However, a growing body of evidence suggests that making errors during learning, far from being hurtful, can be beneficial and foster learning (for a review, see Mera et al., 2022; Metcalfe, 2017). It is, therefore, important to determine the circumstances under which errors enhance memory during learning and to apply this knowledge to adjust practices in educational settings.

A good example of how errors can boost memory is the *pretesting effect* (also known as the *unsuccessful retrieval* and *failed retrieval effect*). Recent studies have shown that introducing tests before being exposed to the learning material can be more beneficial than mere re-reading (Cyr & Anderson, 2018; DiMarco, et al., 2024; Hartley, 1973; Hollins et al., 2023; Kornell et al., 2009; Mera et al., 2022; Pan & Rivers, 2023; Potts & Shanks, 2014; Richland et al., 2009; Seabrooke et al., 2019a, 2019b, 2021a, 2021b; Tanaka et al., 2019; Zawadzka & Hanczakowski, 2019). In this experimental procedure, participants are asked to guess unknown information. Due to a lack of prior exposure to the material, these tests are mostly errorful. However, despite making many errors during learning, memory is boosted, demonstrating how making errors during learning can benefit memory under certain circumstances.

In an early study by Kornell et al. (2009), participants in the pretest condition were presented with a cue word (e.g., pond –?) and asked to guess the target word. Shortly after, they were shown the correct word pair (a weakly semantically related word pair) as corrective feedback (e.g., pond–frog). This condition was compared to an errorless read-only condition, where participants were shown the cue accompanied by the target word and asked to read the word pairs without generating a guess (and thus without making errors) for an equivalent duration to the pretest trials. Despite making numerous errors, participants showed better recall for pretest trials in a subsequent cued recall test. This indicates that pretesting improves recall, even when the initial guesses are incorrect.

Several mechanisms and theoretical frameworks have been proposed to explain how errorful learning benefits memory (Mera et al., 2022). The *Search Set* theory (Carpenter, 2009, 2011; Grimaldi & Karpicke, 2012) proposes that an initial retrieval attempt activates a network of semantically related concepts, including the correct target. When feedback is provided, this activation strengthens the connection between the cue and the correct response, consistent with a spreading activation framework (Collins & Loftus, 1975). Similarly, the *Recursive Reminding* theory (Wahlheim & Jacoby, 2013) emphasizes the role of episodic memory, suggesting that both the error and the feedback are encoded in the same episodic event. By binding the corrective feedback to the prior erroneous response, this co-encoding promotes more robust encoding of the correct answer (as proposed by the *test-potentiated learning* account; Izawa, 1970; Rowland, 2014) and heightens attention to the feedback (as described in the *error monitoring* account; Metcalfe, 2017). Later, retrieving the initial error can reinstate this shared episodic context, facilitating access to the correct information. Furthermore, errors can serve as unique retrieval cues. Incorrect guesses can themselves become associated with the target information, creating additional retrieval pathways (Huelser & Metcalfe, 2012; Metcalfe & Huelser, 2020; Pyc & Rawson, 2010). Finally, the *Prediction Error* framework offers another perspective, focusing on the discrepancy between a learner’s expectations and the actual outcome (e.g., Rescorla & Wagner, 1972). According to this account, when a learner attempts to guess information during a pretest and fails, it shows a prediction error that signals the need for learning and increases attention (Grimaldi & Karpicke, 2012; Metcalfe, 2017). These processes help explain why even high-confidence errors can be particularly memorable when corrected (the *hypercorrection* effect, Butterfield & Metcalfe, 2001; Metcalfe & Miele, 2014). Importantly, these accounts are not mutually exclusive. Rather, they offer complementary perspectives on how errors, effort, and feedback interact during learning to produce lasting memory benefits. Together, these theories align with the *desirable difficulty* framework, which proposes that conditions introducing cognitive effort into the learning process – as occurs during pretesting – enhance learning (Bjork & Bjork, 2011).

Additionally, actively recalling information from memory during learning (i.e., being tested during learning) can promote later correct recall compared to simply re-reading the information. This is known as the *retrieval practice* or *testing effect*, a well-established phenomenon in cognitive and educational sciences that shows the benefits of being tested on newly learned material for later recall (see Roediger & Karpicke, 2006a, for a review, and Rowland, 2014, for a meta-analysis). This effect involves a unique neural network (Marin-Garcia et al., 2021; Van den Broek et al., 2016) and is considered one of the most effective learning techniques (Dunlosky et al., 2013). In a typical retrieval practice experimental procedure, participants are exposed to learning cycles intermixed with test cycles. This group is compared with a control group that studies (reads) the material for the same amount of time, after which a final memory test is completed. Based on prior studies, the testing group usually shows significantly higher correct performance than the control group (e.g., Karpicke & Roediger, 2008; Roediger & Butler, 2011). This effect occurs even if these tests are unsuccessful (Mera et al., 2025b). Indeed, as we have already mentioned, errors, when accompanied by corrective feedback, have beneficial effects on learning (Maraver et al., 2022; Mera et al., 2022; Metcalfe, 2017).

These two learning strategies – retrieval practice and pretesting – share two primary characteristics. First, they involve incorporating tests during the learning phase, either after exposure to the learning material (i.e., retrieval practice) or before exposure (i.e., pretest). Second, they both entail a form of errorful learning, albeit to varying degrees. Retrieval practice allows for errors during the initial test, while pretesting practically encourages errors throughout the learning trial, given that the material being tested is unknown to the participant. In this regard, Wong and Lim (2019) introduced three different approaches to addressing errors: prevention (avoiding errors), permission (allowing errors to occur naturally), and promotion (inducing learners to make errors). From this perspective, retrieval practice can be considered an error-permissive learning method, whereas pretesting promotes error generation by inducing learners to make errors.

While both pretesting and retrieval practice use testing as a learning tool, they may be theoretically distinguished by their timing and primary cognitive influence. Pretesting prepares the learner for encoding by activating prior knowledge. In addition, it encourages the generation of errors, which can enhance attention and improve the processing of subsequent corrective feedback. This preparatory activation and error-correction process has been proposed to optimize how information is initially learned (Mera et al., 2025a, 2025b, 2025c). In contrast, retrieval practice primarily consolidates existing learned information and improves the subsequent retrieval (Pan & Sana, 2021). Thus, the potential advantage of pretesting may lie in its emphasis on optimizing the encoding of *new* information, whereas retrieval practice may be more effective for enhancing memory consolidation and retrieval fluency (e.g., Agarwal et al., 2021).

In the context of the pretesting paradigm, a key concern is the authenticity of errors generated during the pretest. Without prior exposure to the learning material, these errors may reflect mere guesses rather than genuine errors (Potts & Shanks, 2014). This issue is particularly evident with certain types of study materials, such as word pairs, where incorrect responses are determined by the experimenter, and less so with materials like sentence translation (Guzmán-Muñoz, 2020) or trivia questions (Kornell et al., 2009). The absence of initial familiarization with the material raises questions about whether pretest errors truly reflect effortful retrieval attempts. This distinction is important because genuine errors and guesses may engage different emotional and motivational processes (e.g., Tulis et al., 2017; Zhao, 2011), potentially influencing how the material is encoded into memory. Moreover, it predominantly produces incorrect responses, which may have different learning consequences compared to conditions with a more balanced mix of correct and incorrect answers.

By contrast, retrieval practice includes a prior study phase, ensuring that learners engage with the material before retrieval attempts. This design allows for clearer comparisons between the effects of correct responses and genuine errors on final test performance (e.g., Kang et al., 2011). Errors in retrieval practice tend to arise naturally and effortfully during retrieval, resembling real-world educational contexts in which learners work with the material before being assessed. However, retrieval practice presents some limitations with regard to studying errorful learning. One concern is the potential for selection effects that make it difficult to isolate the unique contribution of errorful learning to overall performance.

Addressing these challenges requires studying errorful learning across both paradigms and comparing their respective benefits. Such an integrated approach could clarify how different types of errors (i.e., guesses vs. genuine retrieval failures) shape learning processes and outcomes.

### Pretest versus retrieval practice

The effects of test order on learning sessions have received relatively little attention in the scientific literature. Among the few studies available, Pan and Sana (2021) and Latimier et al. (2019) have both compared the effects of pretesting and retrieval practice, hereafter referred to as pretest and post-test. However, these studies yielded contradictory findings. Pan and Sana (2021) conducted five experiments in which participants were tasked with learning information about a text passage (e.g., information about the planet Saturn). They completed practice questions in a cued recall or multiple-choice format before (pretest) or after (post-test) reading the text passage. Their results revealed that taking a pretest before reading the text passage yielded similar or even greater learning benefits than answering a post-test after reading it. Both types of tests improved memory compared to questions that were not previously tested.

In contrast, Latimier et al. (2019) found the opposite result. In their study, online participants took a multiple-choice practice test with immediate feedback either before (pretest) or after (post-test) reading biology text passages, and were compared to a control condition that read the material for an extended time without testing. On a 1-week delayed final test, both pretesting and post-testing effects were observed. However, in contrast to Pan and Sana’s (2021) findings, the effect was larger for post-testing compared to pretesting conditions.

The contradictory results between Pan and Sana (2021) and Latimier et al. (2019) may be attributed to key methodological differences. First, the studies employed different control conditions. Pan and Sana (2021) compared pre- and post-tested questions to other control questions that were not previously tested. In contrast, Latimier et al. (2019) used a separate reading condition as their control, allowing for matched exposure times across all conditions.

Second, the studies differed in their final test formats. Pan and Sana (2021) used both cued recall and multiple-choice formats, while Latimier et al. (2019) employed only a multiple-choice format. While contradictory results have been reported regarding the influence of the final test format on the retrieval practice effect (e.g., Kang et al., 2007; Little et al., 2012; Rowland, 2014), this difference is unlikely to account for the discrepancy in results between these two studies. Pan and Sana (2021) found no significant effect of test format on final performance, suggesting that in this case, the final test format variation did not substantially impact the results.

Third, an important distinction lies in the nature of the experimental design. Latimier et al. (2019) implemented an online module approach, which can present several potential confounding factors, including reduced experimenter control over participant engagement. In contrast, Pan and Sana (2021) implemented a more controlled experimental design, which is also more comparable to the present experiments. This difference in experimental settings may have influenced participant performance and contributed to the observed discrepancies.

Lastly, the retention intervals varied between the studies. Latimier et al. (2019) implemented a 1-week delay between learning and final testing, whereas Pan and Sana (2021) used both a 5-min0 (Experiments 1, 2, 3, and 5) and a 48-h delay (Experiment 4). The longer delay in Latimier et al.’s study (1-week delay) may have led to more forgetting, potentially enhancing the benefits of post-testing relative to pretesting.

Additionally, both Pan and Sana’s (2021) and Latimier et al.’s (2019) results show the potential effects of pretesting with complex material using text passages. However, it is important to note that prior research has suggested that retrieval-based learning strategies may function differently depending on the complexity of the material (e.g., van Gog & Sweller, 2015; c.f., Karpicke & Aue, 2015). Furthermore, a potential limitation of using text passages is the difficulty in controlling for participants’ prior knowledge, which can influence the effect of pretesting. To mitigate this impact, the present study employed weakly semantically related word pairs, as used in previous research (e.g., Kornell et al., 2009), which were not pre-exposed and help to isolate the effects of previous knowledge.

The mixed results reported by Pan and Sana (2021) and Latimier et al. (2019) highlight the need for further research to clarify the relative effectiveness of pretesting versus post-testing for enhancing long-term memory. The present study aimed to build upon the findings of these two previous studies and address the remaining questions raised by their conflicting results.

### Errorful learning and aging

Most studies on pretesting have used neurologically typical young adults as participants, leaving it unclear whether the effect differs in healthy older adults compared to a younger population. Some research has been conducted on errorful learning with different age groups (Cyr & Anderson, 2012, 2015; Kessel & de Haan, 2003; Metcalfe et al., 2015; Montoro-Membila et al., 2025). Errors have had a negative reputation, suggesting that they might be disadvantageous and should be avoided in cognitive aging (Baddeley & Wilson, 1994; Clare & Jones, 2008; Middleton & Schwartz, 2012). Discriminating errors from targets after errorful learning requires good functioning of episodic memory, which typically declines with age (for a review, see Park & Festini, 2017). In addition, it has been hypothesized that older adults may be more susceptible to memory interference compared with young adults, which could partly explain the differences in episodic memory between these age groups (Biss et al., 2013; Hasher & Zacks, 1988; Jacoby et al., 2001). Thus, people with poorer episodic memory capacity, as might be the case for healthy older adults, may not benefit as much from making errors during learning. Contrary to this expectation, some studies examining the retrieval practice effect in middle-aged to older adults showed that these learners benefited from testing, similar to younger adults (Balota et al., 2006; Meyer & Logan, 2013; Pastötter & Bäuml, 2019; Tse et al., 2010). This suggests that older adults may be able to engage in appropriate encoding processes during the study, yielding similar benefits from retrieval practice to those observed in younger learners.

Concerning the pretesting effect, Cyr and Anderson (2015) studied trial-and-error learning with healthy older adults. Participants had to learn words associated with either semantic categories (e.g., a fruit) or word stems (fl____). In the errorful condition, participants were presented with the cue and had to generate two guesses before receiving corrective feedback. In the errorless condition, participants viewed the cue, and shortly after, the target word. Their findings revealed that experiencing errors during encoding could help to diminish typical age-related declines in episodic memory.

Overall, these preliminary studies suggest that errorful learning strategies, such as pretesting and retrieval practice, may effectively improve memory in older adults. However, more research is needed to fully establish their pedagogical potential. While the pretesting paradigm presented by Kornell et al. (2009) has been extensively studied with neurologically typical young adults (Mera et al., 2022), it has yet to be studied in older populations. This will provide a more comprehensive understanding of the pretest effect across different age groups and help explore the generalizability of pretesting. Given that learning is an essential capacity throughout life, an additional aim of this study is to explore whether the pretest effect extends to healthy individuals over 60 years of age.

### Error types and recovery

Another important issue is whether errors during learning produce proactive interference. The *interference theory* (Melton & Irwin, 1940; Postman & Underwood, 1973) proposes that committing errors increases the distinctiveness of items that compete with the correct answer at retrieval. If this were the case, we would expect to find a perseveration of errors generated during initial pre- or post-tests on the criterion test (i.e., intrusion errors). Knight et al. (2012) explored whether including tests during the learning phase could increase error rates due to more words being active during the study phase. Thus, the committed error could potentially interfere with the correct word given through feedback. As a result, pretested pairs would show more intrusion errors compared to control read-only pairs. Their results showed minimal error intrusion during the final test for pretested items (< 10%). Further supporting this, Kliegl et al. (2023) found that the pretesting effect remains robust even in the presence of competing information. Their study suggests that pretesting facilitates the integration of errors into a learning framework that promotes correct retrieval, rather than increasing interference. This aligns with previous research showing that taking a test after exposure to learning material (e.g., retrieval practice) can protect the tested material from interference caused by subsequently studied, and potentially overlapping, material (Halamish & Bjork, 2011; Potts & Shanks, 2012). As a result, tests seem to provide protection against interference, whether administered before (pretest) or after (retrieval practice) exposure to the material.

Similarly, Mera et al., (2025a, 2025b, 2025c) studied intrusion errors in an attempt to reproduce the so-called “derring effect,” where deliberately generating errors, even when the correct answer is known, appears to substantially improve memory (Wong & Lim, 2022). Apart from the credibility of the effect, even when participants were asked to commit errors deliberately, only 4% of the incorrect test responses were intrusion errors. Considering this previous research, examining the intrusion errors on the final cued-recall tests can provide additional insights into how pretesting and retrieval practice impact memory.

### Metamemory

Metamemory refers to the knowledge about our memory function, including estimations of our learning capacity and strategies that can be used to improve it (Flavell & Wellman, 1977; Metcalfe & Shimamura, 1994). Learners’ intuitive beliefs about their learning often do not align with the actual outcomes. Learners typically tend to think that errors are harmful (Huelser & Metcalfe, 2012; Yang et al., 2017), which can lead to avoiding them during learning (Tulis, 2013). For example, in studies by Potts and Shanks (2014) and Seabrooke et al., (2019a, 2019b), participants were asked to estimate their likelihood of remembering word pairs. In these studies, participants gave lower ratings to the word pairs from the errorful learning condition. To explore the metamemory of participants, they examined their degree of awareness about the impact of errors on their learning. To this end, participants in this study were asked to predict the material they got correct and to show their beliefs about the benefits of errors for their learning.

### The present study

The present study aimed to expand our understanding of the potential effectiveness of pretesting versus post-testing in enhancing long-term memory, focusing on the impact of error generation during learning. Building upon previous research, our investigation addresses several key objectives: to analyze the influence of test order (before or after learning) on subsequent correct recall using semantically related word pairs to mitigate the impact of prior knowledge of the material. Furthermore, we administered the final test immediately after a distractor task to further isolate the effects of retention interval differences in the final test. Finally, Experiment 3 explores the generalizability of pretesting effects on healthy older adults, shedding light on potential age-related differences in errorful learning. Through these experiments, we aimed to elucidate the effects of pretesting and post-testing using word pairs with no prior exposure and explore age-related differences. The present study aimed to provide a more comprehensive understanding of the conditions under which each strategy is most effective for enhancing long-term memory.

## Experiment 1

In Experiment 1, participants learned weakly semantically related word pairs (e.g., seal–ball) under one of three conditions: (1) a pretest condition, where they attempted to guess targets before seeing the correct pairs; (2) a post-test condition, involving retrieval practice after study; or (3) a copy condition, where they passively restudied pairs without testing. This design allowed a direct comparison of errorful learning (pre- and post-testing) versus errorless learning (copying).

Based on prior robust evidence for retrieval practice (e.g., Roediger & Karpicke, 2006a) and pretesting effects (e.g., Kornell et al., 2009), we expected that both pretest and post-test groups would outperform the copy group on the final cued-recall test, reflecting the mnemonic advantage of errorful learning. We did not anticipate significant differences in recall accuracy between pretest and post-test conditions, as both involve active retrieval – of either initial guesses (pretest) or previously studied material (post-test).

While we did not have strong predictions regarding reaction times (RTs), prior research suggests that errors tend to slow down subsequent responses (e.g., Rabbitt & Rodgers, 1977) and require longer processing times to retrieve the correct answer in a final test (Mera et al., 2025b). Given this, it was anticipated that learning conditions involving errors (i.e., pre- and post-test) would lead to longer response times compared to the errorless copy condition. Additionally, we expected that participants in the pretest condition would exhibit longer response times during the final test than those in the post-test condition, as their exposure to more errors during learning might result in distinct retrieval processes. Regarding proactive interference, we predicted that all testing conditions would yield a low proportion of intrusion errors, indicating minimal interference from previous errors. Finally, based on prior research on errorful learning and metamemory (e.g., Potts & Shanks, 2014; Seabrooke et al., 2019a, 2019b), we anticipated that participants would be unaware of the beneficial impact of errors on their learning, underestimating the advantages of errorful learning strategies.

### Method

#### Participants

Ninety-three students from the University of the Basque Country UPV/EHU were randomly allocated to three groups: The *pretest* group (n = 31; 25 females, six males; *M*_age_ = 19.58, *SD*_age_ = 3.6 years), the post-test group (n = 31; 23 females, six males, two others; *M*_age_ = 19.39, *SD*_age_ = 3.9 years) and a control *copy* group, which did not involve testing (n = 31; 29 females, two males; *M*_age_ = 18.81, *SD*_age_ = 1.74 years). A minimum sample size of 29 participants per group was determined based on a power analysis (G*Power; Faul et al., 2007), setting α to.05 and β to.20, to detect the overall effect size of g = 0.75 for the standard subsample found in Chan et al.’s (2018) meta-analytic review.

The sample was primarily composed of psychology students, typically including a higher proportion of females, which accounts for the gender sample bias in our study. All participants confirmed being native Spanish language users and reported no diagnosed developmental disorders (e.g., attention-deficit hyperactivity disorder (ADHD), dyslexia), psychological or psychiatric disorders, or use of medication that could affect task performance. Before participation, volunteers consented to participate in the study following the regulations set by the Ethics Committee for Research and Teaching (CEID/IIEB) of the UPV/EHU (M10-2019–215).

#### Design

The experiment adopted a between-subjects design with a main independent variable, learning condition, which had three levels: pretest, post-test, and copy. The dependent variable was the number of correctly recalled target words in a cued-recall final test. A between-subjects design was favored over a within-subjects design to ensure that each participant’s performance in their assigned condition was not influenced by exposure to other conditions (i.e., carryover effects), which is particularly crucial in learning and memory studies.

#### Material

The study material comprised a list of 108 weakly semantically related Spanish word pairs (e.g., seal–ball). These pairs were chosen from the NALC – *Normas de Asociación Libre en Castellano de la Universidad de Salamanca* (Fernández et al., 2010). Following Kornell et al.’s selection criteria (2009), we specifically selected pairs with a forward cue-to-target strength between.01 and.054. This means that when the cue word was presented, the target word was the first association for 5% of the participants. All the words were nouns with at least four letters and showed no significant differences in imaginability and concreteness values (*p* >.05; EsPal repository, Duchon et al., 2013).

#### Procedure

All participants completed three experimental phases: a learning phase, a distractor task phase, and a final test phase. In addition, three-word pair practice trials preceded each phase, and participants were encouraged to address any questions they might have during that time.

##### Learning phase

The order of this phase varied according to the learning condition group. The Pretest group started with an initial cued-recall test in which they were asked to guess the targets for each cue individually, one at a time (e.g., seal –?). After each guess, they were given corrective feedback for 3 s (i.e., seal–ball). Following this, they completed a study phase in which they first read each word pair for 3 s. Then, the target word disappeared, and they were instructed to type the word that had been shown earlier. Typed responses (rather than passive reading) were used to promote deeper cognitive processing of the information (Craik & Tulving, 1975). This enabled us to control for the generation effect, where actively self-generated information is better remembered than passively read information (Slamecka & Graf, 1978), while maintaining a less cognitively active comparative condition.

The Post-test group followed a different order, starting with the study session and later completing the initial cued-recall test with corrective feedback + copy. This procedure ensured the same amount of studying and testing for both pretest and post-test groups. Lastly, the control (or Copy) group completed two consecutive study sessions. In each session, they read each word pair for 3 s and then copied the target word after being given the cue.

##### Distractor task phase

Participants completed a 5-min Continuous Performance Test in which letters appeared on the screen in rapid succession. Their task was to press the space bar only when they encountered a target letter (e.g., “K”), and they were instructed to refrain from responding to any other letters presented.

##### Final test phase

Participants from all three groups completed the same cued-recall test. They were shown the study phase cues and asked to recall the corresponding targets correctly. No corrective feedback was given during this final phase.

The final test was self-paced, allowing participants to take as much time as needed to provide their responses. Additionally, participants were not allowed to leave their answers blank (i.e., no omission errors). If they could not recall or guess the correct target, they were encouraged to type the first word that came to mind. This approach was designed to analyze the impact of commission errors.

After completing the final test, participants responded to two metacognitive questions using a slider ranging from 0 to 100%. First, they were asked to predict the percentage of material they believed they had answered correctly on the final test. Then, they responded to the question: “What is more effective for your learning process, experiencing correct answers or making errors?” Participants used a slider with “correct answers” on the left and “errors” on the right to indicate their response.

**Fig. 1:**
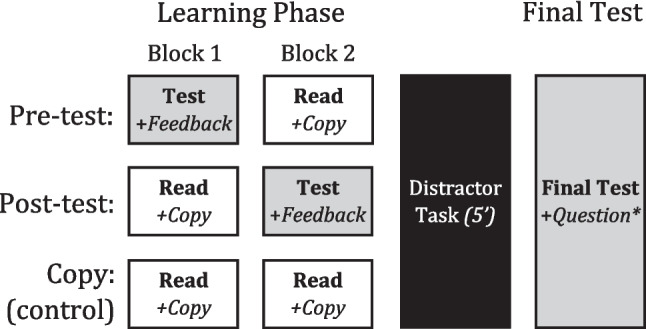
Procedure for Experiment 1

The experiment was conducted in a multi-station laboratory, allowing up to five participants to be tested at the same time. The material was presented in white on a black background on a 1,680 × 1,050-pixel resolution computer. The open-source software “PsychoPy” (v.1.9.1) was used for stimulus presentations and data collection (Peirce et al., 2019), which allows the recording of RTs precisely (Bridges et al., 2020). At the end of the experiment, participants were debriefed and thanked for their participation. The entire experiment lasted approximately 40 min (Fig. 1).

### Results

#### Scoring

All analyses were conducted using RStudio (R Core Team, 2023). Responses typed by participants in the initial and final cued-recall test were recorded and evaluated for accuracy. The computer program scored participants’ responses as correct if the typed word matched the correct target. Only 5.08% of pretested trials were correctly guessed in Experiment 1, 4.63% in Experiment 2, and 5.61% in Experiment 3. To isolate the effects of erroneous guesses on subsequent recall performance, it is standard practice in the pretesting literature to remove items correctly answered during pretesting (see Kornell et al., 2009, for a discussion on this issue). However, in alignment with the methodologies used in Pan and Sana (2021) and Latimier et al. (2019), we opted not to apply this exclusion criterion.

Before analyzing the data, typographical errors were reviewed. In the few cases where the word was not accented correctly (e.g., “inyeccion” vs. “inyección”), it was coded as correct. The computer program also converted all responses to lowercase before scoring accuracy. We also checked for accidental spaces in the participants’ responses during data processing (e.g., “inyección_” or “inye_cción”). Any accidental spaces were removed, and accuracy was re-scored for those trials.

We also investigated the types of errors that participants made in each learning condition. Following previous studies (e.g., Knight et al., 2012; Mera et al., 2025c), participants’ errors were categorized into three types: (a) *commission errors* – inadequate or incorrect responses that were different from participants’ errors in the learning phase; (b) *confusions* – responses that provided the target of a different cue; and (c) *intrusions* – responses that repeated the same errors that participants made during the learning phase (only possible in pretest and post-test conditions). Note that cued-recall tests were forced responses. Thus, no omission errors were allowed for this experiment.

Reaction times (RTs) at the final test were measured by calculating the seconds between the cue presentation and the first key press in the response. RT trials above two standard deviations (> 2*SD*) of the participant’s mean or faster than 100 ms were adjusted to the participant’s mean RT (e.g., Notebaert et al., 2009). Based on these criteria, 5.22% of trials in Experiment 1, 5.25% in Experiment 2, and 5.44% in Experiment 3 were adjusted. Note that these criteria are used solely to clean the RT data by addressing unusually late responses. Importantly, our RT analysis remains largely unchanged even without these adjustments. However, we opted to apply them to present a clearer dataset. For transparency, the original RT data and analyses are available on the Open Science Framework (see *Data availability*).

#### Cued-recall performance

**Fig. 2:**
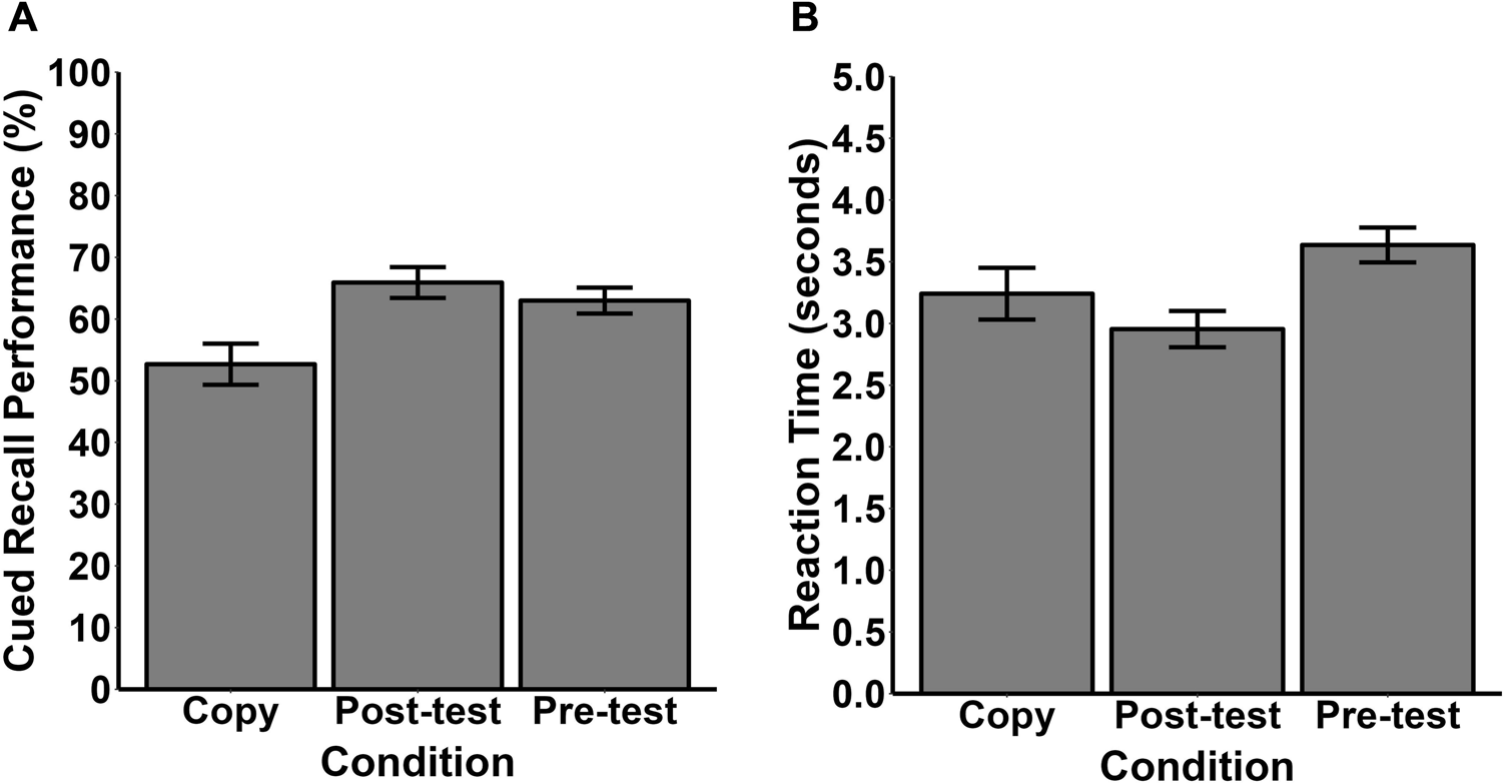
Results of Experiment 1 comparing Pretest, Post-test, and Copy groups. (**A**) Mean percentage of cued-recall test accuracy. (**B**) Reaction time (in seconds) at the final test. Error bars represent standard error of the mean (± 1 SEM)

A one-way between-subjects ANOVA revealed a significant learning group (pretest, post-test, copy) effect on cued recall performance, *F*(2, 90) = 6.67, *p* =.002, ${\eta }_{g}^{2}$ =.13 (see Fig. 2A). Post hoc *t*-test comparisons with a Bonferroni correction of* p* =.0167 (=.05/3) indicated that participants in the post-test group (*M* = 65.92, *SD* = 13.9) performed significantly better than those in the copy group (*M* = 52.69, *SD* = 18.55), *t*(60) = 3.18, *p* =.002, *d* = 0.81. Similarly, the pretest group (*M* = 62.99, *SD* = 11.69) also outperformed the copy group, *t*(60) = 2.62, *p* =.01, *d* = 0.66. No significant differences were found between the pretest and post-test groups, *t*(60) = 0.9, *p* =.37, *d* = 0.23.

#### Error-type analysis

To examine the type of errors (commission, confusion, intrusion) made by participants in each learning condition (pretest, post-test, copy), we conducted separate one-way between-subject ANOVAs for each error type. We did not conduct a 3 × 3 (learning condition × error type) one-way ANOVA because intrusion error types only applied to the pretest and post-test groups. The same approach was adopted for the other experiments in the study. First, regarding *commission errors*, the ANOVA revealed a significant main effect of learning condition, *F*(2, 90) = 61.45, *p* <.001, ${\eta }_{g}^{2}$ =.58. Post hoc *t*-test comparisons (Bonferroni correction of *p* =.0167) revealed that participants in the copy condition (*M* =.82, *SD* =.80) made significantly more commission errors than those in both the pretest, (*M* =.60, *SD* =.12), *t*(60) = 8.31, *p* <.001, *d* = 2.11, and the post-test learning conditions, (*M* =.55, *SD* =.09), *t*(60) = 11.78, *p* <.001, *d* = 2.99. No statistical differences were found between the pretest and post-test conditions, *t*(60) = 1.78, *p* =.08, *d* = 0.45. For *confusion errors*, no significant differences were found between the pretest (*M* =.17, *SD* =.07), post-test (*M* =.19, *SD* =.08), and copy conditions (*M* =.18, *SD* =.08), *F*(2, 90) = 0.45, *p* =.64, ${\eta }_{g}^{2}$ =.01. Most incorrect responses on the final tests for all conditions were commission errors (*p* <.05). Importantly, for *intrusion errors*, the rates were comparable between the pretest (*M* =.23, *SD* =.09) and post-test learning conditions (*M* =.26, *SD* =.11), with no significant difference observed, *t*(60) = 1.27, *p* =.21, *d* = 0.32. This indicates that errors made during learning (both before and after exposure to the learning material) did not cause significant interference. Table 1 displays the mean proportions and standard deviations for each error type.

**Table 1:** Proportion of commission, confusion, and intrusion errors at the final test

	Experiment 1			Experiment 2				Experiment 3	
Error type	Pretest	Post-test	Copy	Pretest	Post-test	CTC	Copy	Pretest	Copy
Commission	.60 (.12)	.55 (.09)	.82 (.08)	.61 (.18)	.53 (.14)	.53 (.14)	.85 (.10)	.62 (.15)	.85 (.08)
Confusion	.18 (.07)	.19 (.08)	.18 (.08)	.19 (.11)	.25 (.14)	.25 (.12)	.15 (.10)	.19 (.07)	.15 (.08)
Intrusion	.22 (.09)	.26 (.11)	*––*	.20 (.13)	.22 (.13)	.22 (.13)	*––*	.20 (.12)	*––*

Intrusion errors – incorrect test responses that repeated the learners’ errors generated during the learning phase – did not apply to the copy condition. Standard deviations are given in parentheses

#### Reaction times

RTs at the final test were analyzed using a one-way ANOVA, revealing a significant difference between learning conditions, *F*(2, 90) = 4.11, *p* =.02, ${\eta }_{g}^{2}$ =.08 (see Fig. 2B). Post hoc *t*-test comparisons (Bonferroni correction of *p* =.0167) revealed that participants in the pretest condition (*M* = 3.64, *SD* = 0.79) were slower to respond than those in the post-test condition (*M* = 2.95, *SD* = 082), *t*(60) = 3.34, *p* =.001, *d* = 0.85. However, RTs did not differ significantly between the pretest and copy conditions (*M* = 3.24, *SD* = 1.17), *t*(60) = 1.56, *p* =.12, *d* = 0.39, or between post-test and copy conditions, *t*(60) = 1.12, *p* =.27, *d* = 0.28.

#### Metacognitive questions

Before concluding the experiment, participants were asked two consecutive metacognitive questions: (1) “What percentage of correct answers do you think you got in the final test?” and (2) “What is more beneficial for learning: experiencing errors or correct answers?”. First, participants rated having a lower performance than their actual performance in both the pretest (39.58 vs. 62.99), *t*(60) = 6.22, *p* <.001, *d* = 1.58, and post-test conditions (45.19 vs. 65.92), *t*(60) = 4.67, *p* <.001, *d* = 1.19. In contrast, in the copy condition, rated and actual performance did not differ significantly (47.03 vs. 52.69), *t*(60) = 1.12, *p* =.27, *d* = 0.29. Thus, participants were more accurate in rating the impact of studying than the influence of a learning strategy involving testing.

Additionally, a one-way ANOVA revealed a statistically significant difference among the three learning condition groups regarding the effectiveness of correct answers versus errors in their learning, *F*(2, 90) = 4.40, *p* =.015, ${\eta }_{g}^{2}$ =.09. Participants in the copy condition (*M* = 64.00%; *SD* = 15.89) rated experiencing errors as more beneficial for their learning compared to those in the pretest learning condition (*M* = 48.03%; *SD* = 23.79; *t*(60) = 3.11, *p* =.003, *d* = 0.79), with a Bonferroni correction of *p* =.0167. However, no significant differences were found compared to the post-test condition (*M* = 59.58%, *SD* = 24.89; *t*(60) = 0.83, *p* =.41, *d* = 0.21), and between pretest and post-test conditions, *t*(60) = 1.87, *p* =.07, *d* = 0.48 [copy > pretest ≈ post-test]. Thus, participants in errorful learning conditions (pretest and post-test) underestimated their performance, indicating a lack of metacognitive awareness of their benefits.

### Discussion

This experiment aimed to explore the impact of error generation during learning and, more specifically, the influence of test order on memory. Overall, both pre- and post-test groups showed superior cued recall compared to the control condition. This finding aligns with previous research highlighting the beneficial effects of testing on memory retention, regardless of whether testing occurs after (Karpicke & Roediger, 2008; Roediger & Butler, 2011) or before learning (e.g., Kornell et al., 2009; Seabrooke et al., 2021a, 2021b, 2022). However, no significant difference in correct recall was observed between the pre- and post-test conditions, suggesting that the testing order did not significantly impact memory performance.

Although we did not find significant differences between pretesting and retrieval practice in our study, it is important to further consider the distinct processes that activate these two learning procedures. One possible differentiation between pretesting and retrieval practice relates to differences in cognitive effort across conditions. In the post-test condition, participants engaged in active retrieval of the studied material, which likely imposed greater cognitive effort during the learning phase and may have facilitated more effective memory consolidation. By contrast, in the pretest condition, participants made guesses before studying the correct information, which might not have triggered the same level of cognitive engagement during initial exposure.

Another possible dissimilarity between pretesting and retrieval practice may be related to test-potentiated learning, which occurs when unsuccessful retrieval attempts enhance the effectiveness of subsequent encoding opportunities (Arnold & McDermott, 2013). Test-potentiated learning can manifest differently in pretest and post-test conditions due to several factors. In our Experiment 1, taking a pretest may alter how participants encode the information during the subsequent study session. This pretest could change participants’ approach to learning the material, potentially activating relevant knowledge structures and making subsequent encoding more effective. In contrast, the post-test condition does not offer the same opportunity since the test occurs after studying.

Our analysis of error types revealed that commission errors (novel incorrect responses) were the most prevalent across all conditions, consistent with previous work showing that learners rarely persist with their initial errors when corrective feedback is provided (Knight et al., 2012). This predominance of commission over intrusion errors suggests that participants engaged in active error correction rather than simply recalling their initial erroneous responses. Importantly, the rate of intrusion errors, which can be taken to indicate some degree of interference from previous errors, did not significantly differ between the pretest and post-test conditions. Furthermore, the low rate of intrusion errors in our experiment suggests that pretesting does not induce significant proactive interference. This is consistent with recent evidence that pretesting can protect the tested material from interference (Kliegl et al., 2023). This suggests that experiencing errors during learning, regardless of whether they occurred before or after the test, did not lead to significant interference during memory retrieval. The proportion of confusion errors was similar across the pretest, post-test, and copy conditions.

On the final test, participants in the pretest condition exhibited slower RTs compared to those in the post-test condition. This finding suggests potential differences in cognitive processing strategies between the pre- and post-test conditions. During the learning phase, the post-test condition likely required more effortful retrieval processes, as participants were actively involved in retrieving previously studied material. In contrast, participants in the pretest condition were simply asked to guess the correct information without prior exposure. This difference in learning approach may have influenced the cognitive effort required during retrieval in the final test. Specifically, the pretest condition may have required more effortful retrieval processes at the final test because it was the first time the participants retrieved that information. Conversely, the post-test condition may have involved a more fluent retrieval process due to the repeated exposure and retrieval practice received by participants during the learning phase. Interestingly, no significant difference in RTs was observed between pretest and post-test conditions compared to the copy condition. This could indicate that the errorless learning condition activated more automatic processes, potentially explaining the higher response rapidity and the absence of differences in RTs among the copy, pretest, and post-test conditions.

Our results also revealed discrepancies between participants’ perceived performance and actual performance, particularly in the errorful learning conditions (pretest and post-test). Participants tended to underestimate their performance in these conditions, indicating a lack of metacognitive awareness regarding the benefits of errorful learning. In addition, they were more accurate in estimating the effect of studying on their performance, possibly because they are more accustomed to applying this learning strategy in their everyday experiences.

The absence of a significant difference in correct recall between the pretest and post-test conditions in our study differs from the findings of Latimier et al. (2019) but aligns with Pan and Sana (2021), extending their findings to a different set of materials (semantically related word pairs). In Pan and Sana’s first experiment, both pretesting and post-testing improved memory performance compared to the control condition. The discrepancy in these findings (Latimier et al., 2019, vs. Pan & Sana, 2021, and our work) could be attributed to the interval between the initial error and final tests. Latimier et al. (2019) introduced the final test after a 1-week delay, showing a beneficial effect in the post-test condition but not in the pretest condition, whereas there was a retention interval of 5 min in the present study. Research on retrieval practice has consistently shown that increasing the time between the practice test and the final test amplifies the effect (e.g., Dunlosky et al., 2013; Roediger & Karpicke, 2006b). Most studies on pretesting have employed short retention intervals, administering the final test shortly after the learning phase (e.g., Grimaldi & Karpicke, 2012), with some exceptions that administered the final test after a 1-day delay (Kornell, 2014; Kornell et al., 2009). Therefore, a longer retention interval could reveal more pronounced differences between pretest and post-test conditions.

Additionally, the design of Experiment 1 may have caused differences in the retention interval. In the pretest condition, the initial test is administered in Block 1 (see Fig. 2), while in the post-test condition, it is conducted in Block 2. As a result, the retention interval between the initial pretest and the final test is longer than that between the post-test and the final test. This difference in retention intervals may counteract the encoding benefits of the pretest, potentially leading to mixed effects on final test performance.

## Experiment 2

To further explore the potential impact of retention intervals within our experimental design, Experiment 2 investigates whether an additional opportunity to encode information following an errorful test can enhance recall performance. We introduced a “copy-test-copy” (CTC) learning condition, allowing participants another chance to study the material after the initial errorful test.

Prior research has shown that repeated exposure and elaboration can strengthen memory retention (e.g., Karpicke & Roediger, 2008), suggesting that supplementing retrieval practice with an additional study phase may amplify the benefits of testing. Thus, we predicted that participants in this enhanced encoding condition would exhibit superior recall compared to those in the standard copy condition. Furthermore, if the primary mechanism underlying the pretesting and post-testing effects is increased, encoding opportunity rather than the moment of the test itself, we expected performance in the additional encoding condition (i.e., CTC) to be higher than in the post-test condition and comparable to the pretest condition. Similar to Experiment 1, we predicted that all testing conditions would show a low proportion of intrusion errors, thus, low interference from previous errors. This design also enables us to compare how different retention intervals between initial and final tests influence performance, providing deeper insights into the temporal aspects of test-potentiated learning.

### Method

#### Participants

The participants were 123 students from the University of the Basque Country UPV/EHU, who were randomly allocated to four groups: the pretest group (n = 31; 26 females, four males, one other; *M*_age_ = 18.61, *SD*_age_ = 0.76 years), the post-test group (n = 31; 26 females, five males; *M*_age_ = 18.68, *SD*_age_ = 0.7 years), the copy-test-copy (CTC) group (n = 30; 24 females, five males, one other; *M*_age_ = 18.57, *SD*_age_ = 0.86 years) and the copy group (n = 31; 23 females, eight males; *M*_age_ = 20.39, *SD*_age_ = 6.01 years).

#### Design

The experiment adopted a between-subjects design. The main independent variable was the learning condition, which had four levels: pretest, post-test, copy-test-copy (CTC), and copy. The dependent variable was the number of correctly recalled target words in a cue-recall final test.

#### Procedure and materials

**Fig. 3:**
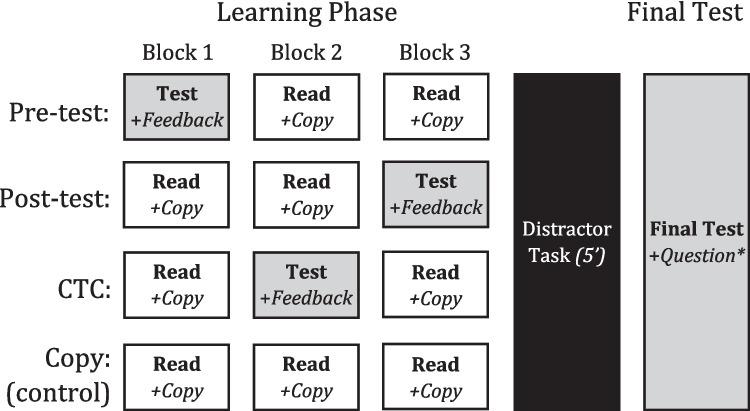
Procedure for Experiment 2

The main difference from the previous experiment concerned the order and extension of the learning phase, with the inclusion of an extra learning block. The rest of the experimental procedure and materials were identical to Experiment 1. The extra learning block was added to study the influence of providing an additional encoding opportunity, following Pan and Sana (2021). Thus, participants were assigned to four groups: the pretest group started with an initial cued-recall test followed by corrective feedback, and then completed two consecutive study sessions. The post-test group started with two consecutive study sessions and then performed the cued-recall test. The copy-test-copy (CTC) group completed one study session before and another after the cued-recall test. In addition, the copy group completed three consecutive study sessions. Following the learning phase, all groups performed the same final cued-recall test after a 5-min distracting task. This procedure ensured the same amount of studying and testing for the pretesting, post-testing, and CTC groups, thus this procedure controlled the duration of each phase to ensure consistency across conditions but manipulated the timing of testing in the learning phase (Fig. 3).

### Results

#### Cued-recall performance

**Fig. 4:**
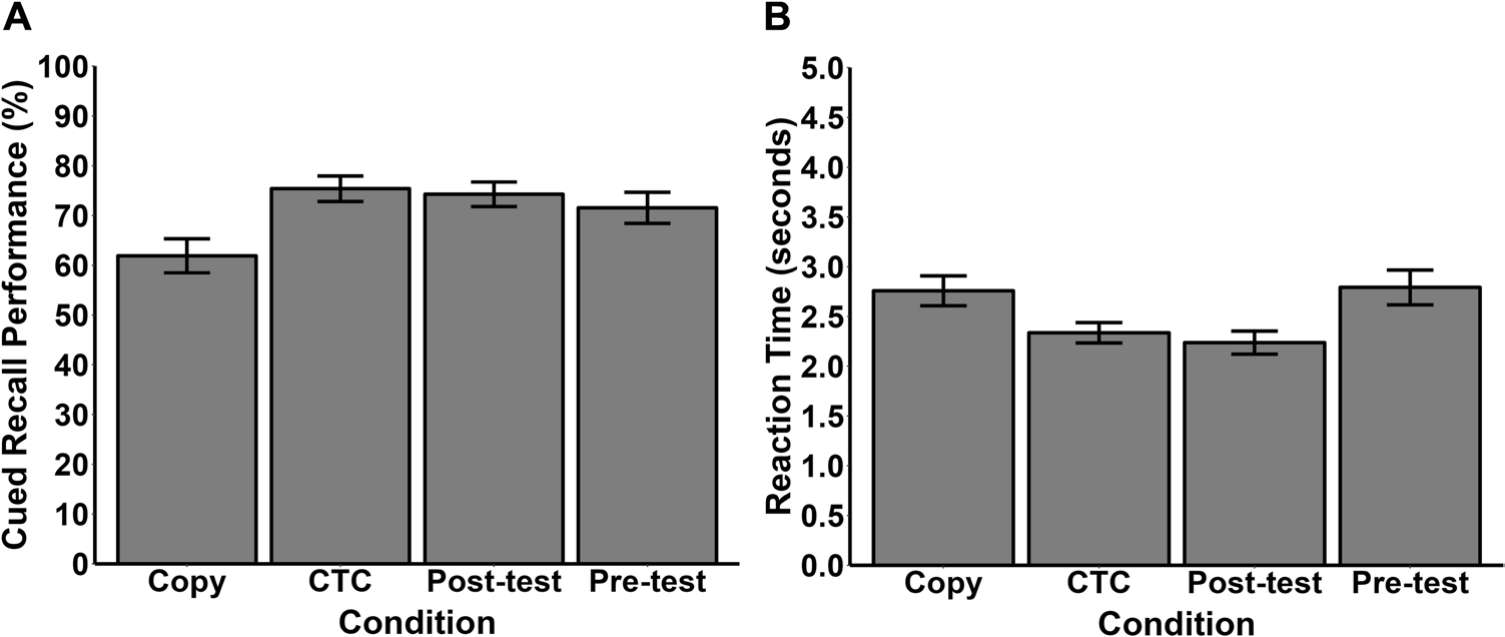
Results of Experiment 1 Comparing the Pretest, Post-test, and Copy groups. (**A**) Mean percentage of cued-recall test accuracy. (**B**) Reaction time (in seconds) at the final test. Error bars represent standard error of the mean (± 1 SEM)

A one-way between-subjects ANOVA revealed a significant main effect of learning condition (pretest, post-test, CTC, copy) on cued-recall performance, *F*(3, 119) = 4.42, *p* =.006, ${\eta }_{g}^{2}$ =.10 (see Fig. 4A). Participants in the copy condition (*M* = 61.89, *SD* = 19) exhibited lower performance compared to the post-test group (*M* = 74.25, *SD* = 13.73), *t*(60) = 2.94, *p* =.005, *d* = 0.75, and the CTC group (*M* = 75.37, *SD* = 14.08), *t*(59) = 3.14, *p* =.003, *d* = 0.80. Although the difference between pretest (*M* = 71.54, *SD* = 17.3) and copy was significant at *t*(60) = 2.09, *p* =.04, *d* = 0.53, this did not survive the Bonferroni-corrected threshold of *p* <.0125 (=.05/4). No significant differences were observed among the test conditions (*p* >.05).

#### Error-type analysis

We examined the types of errors made by participants in each learning condition (see Table 1) using a one-way between-subjects ANOVA. This analysis revealed a main effect of learning condition (pretest, post-test, copy, CTC) on the proportion of *commission* errors made, *F*(3, 119) = 33.67, *p* <.001, ${\eta }_{g}^{2}$ =.46. Commission errors in the copy group (*M* =.85, *SD* =.10) were higher than those made by participants in the pretest (*M* =.61, *SD* =.18),* t*(60) = 6.47, *p* <.001, *d* = 1.64, post-test, (*M* =.53, *SD* =.15), *t*(60) = 9.98, *p* <.001, *d* = 2.53, and CTC learning conditions, (*M* =.54, *SD* =.14), *t*(60) = 10.58, *p* <.001, *d* = 2.71. No significant differences were found between the pretest, post-test, and CTC groups (*p* >.05).

Similarly, a one-way between-subjects ANOVA revealed a main effect of learning condition on the proportion of *confusion* errors, *F*(3, 119) = 5.38, *p* =.002, ${\eta }_{g}^{2}$ =.12. Participants in the CTC group made a higher proportion of confusion errors (*M* =.25, *SD* =.12) compared to the pretest condition (*M* =.18, *SD* =.11), *t*(59) = 2.1, *p* =.04, *d* =.54, and the copy condition (*M* =.15, *SD* =.10), *t*(60) = 3.55, *p* <.001, *d* =.91, but not the post-test condition (*M* =.25, *SD* =.14), *t*(59) = 0.05, *p* =.96, *d* = 0.01. Participants in the post-test condition also showed a higher proportion of confusion errors than those in the copy condition, *t*(60) = 3.26, *p* =.002, *d* =.83. However, a Bonferroni correction of *p* <.0125 (=.05/4) indicated that the difference between the CTC and pretest comparison (*p* =.04) fell short of significance. No other group comparisons were significant (*p* >.05).

The majority of final test incorrect responses across all learning conditions were commission errors (*p* >.05). Importantly, the rate of *intrusion* errors was similar in the pretest (*M* =.20, *SD* =.13), post-test (*M* =.22, *SD* =.13), and the CTC learning conditions (*M* =.22, *SD* =.13), *F*(2, 89) = 0.24, *p* =.79, ${\eta }_{g}^{2}$ =.01. This indicates that experiencing errors during learning, whether before or after exposure to the learning material, did not cause significant interference.

#### Reaction times

Analysis of RTs revealed a main effect of learning condition, *F*(3,119) = 4.23, *p* =.007, ${\eta }_{g}^{2}$ =.10 (see Fig. 4B). Participants in the pretest condition (*M* = 2.79, *SD* = 0.97) showed higher RTs compared to those in the post-test (*M* = 2.24, *SD* = 0.64; *t*(60) = 2.65, *p* =.01, *d* = 0.67) and CTC conditions (*M* = 2.34, *SD* = 0.56; *t*(59) = 2.24, *p* =.03, *d* = 0.57. The copy condition (*M* = 2.76, *SD* = 0.83) also showed higher RTs than the post-test (*t*(60) = 2.76, *p* =.008, *d* = 0.70) and CTC conditions *t*(59) = 2.32, *p* =.02, *d* = 0.60. No significant differences were found between the pretest and copy groups (*t*(60) = 0.15, *p* =.88, *d* = 0.04) and between the post-test and CTC groups (*t*(60) = 0.64, *p* =.53, *d* = 0.16). Bonferroni correction *p* <.0125 (=.05/4) indicated that the pretest versus post-test comparison (*p* =.01) did not survive correction, and the copy versus CTC comparison (p =.02) was not significant either after correction. Thus, participants in both the pretest and copy conditions responded significantly slower than those in both the post-test and CTC conditions.

#### Metacognitive questions

Participants rated their performance lower than their actual performance in the pretest (53.97 vs. 71.53), *t*(60) = 3.76, *p* <.001, *d* = 0.96, post-test (52.26 vs. 74.25), *t*(60) = 4.58, *p* <.001, *d* = 1.16, and CTC conditions (53.57 vs. 75.37), *t*(58) = 5.28, *p* <.001, *d* = 1.36. In contrast, in the copy condition, participants’ rated and actual performance did not differ significantly (54.26 vs. 61.89), *t*(60) = 1.69, *p* =.10, *d* = 0.43. Thus, participants underestimated their performance in learning conditions that required testing (pretest, post-test, and CTC) and were more accurate in predicting their copying performance. Additionally, a one-way ANOVA revealed a statistically significant difference among the four learning condition groups regarding the effectiveness of correct answers versus errors in their learning, *F*(3, 119) = 3.30, *p* =.02, ${\eta }_{g}^{2}$ =.08. The post-test condition (*M* = 64.58%, *SD* = 24.38) rated experiencing errors as more beneficial for their learning compared to both the CTC (*M* = 51.4%, *SD* = 21.99; *t*(59) = 2.21, *p* =.03, *d* = 0.57) and the pretest condition (*M* = 48.51%, *SD* = 26.15; *t*(60) = 2.50, *p* =.01, *d* = 0.64), but not compared to the copy condition (*M* = 59.13%, *SD* = 15.54; *t*(60) = 1.05, *p* =.30, *d* = 0.64). The copy condition rated errors as being beneficial for their learning marginally higher compared to the pretest condition (*t*(60) = 1.94, *p* =.06, *d* = 0.49), but not compared to the CTC group, *t*(59) = 1.59, *p* =.12, *d* = 0.41. Finally, no significant differences were found between the CTC and pretest learning conditions, *t*(59) = 0.47, *p* =.64, *d* = 0.12 [post-test ≥ copy ≥ CTC ≥ pretest]. However, the difference between post-test and CTC (*p* =.03) fell short after applying the Bonferroni correction (adjusted threshold of *p* =.0125, 0.05/4).

### Discussion

Experiment 2 aimed to investigate the influence of providing an additional encoding opportunity after errorful testing on subsequent recall performance compared with pretesting, post-testing, and a control (copy) condition. Concerning cued recall performance, participants in the copy condition exhibited lower performance compared to the pretest, post-test, and CTC groups (although the difference between the copy and pretest conditions did not remain statistically significant after applying the Bonferroni correction). This finding suggests that simply copying the material without engaging in errorful testing may result in poorer recall compared to other learning conditions, replicating the results of Experiment 1. Our results are consistent with previous studies demonstrating that including tests as learning interventions can enhance memory compared to passive learning strategies (e.g., Rowland, 2014).

Our results suggest no significant differences in correct recall among the pretest, post-test, and CTC groups, indicating that providing an additional encoding opportunity after errorful testing (CTC condition) did not significantly impact recall performance compared to errorful testing alone. Pan and Sana’s (2021) Experiment 5 introduced a similar condition (read-test-read) where a post-test occurred after the first reading of the text passage and before the second reading. Their findings revealed that both the pretest and the read-test-read conditions yielded superior performance compared to post-test questions, indicating that testing alters the encoding of subsequent information. Thus, Pan and Sana (2021) demonstrated an advantage for pretesting and CTC (or read-test-read in their study) over conventional post-testing. We did not find such an advantage, even when an extra encoding opportunity was provided. Additionally, participants might perceive the CTC condition as more effortful, which we did not assess. Future research could explore participants’ subjective experiences related to effort in each condition. Specifically, this could be done by incorporating measures of perceived difficulty, to provide a more comprehensive understanding of the cognitive demands associated with the CTC condition and how these perceptions might relate to learning outcomes.

The examination of error types rev0ealed that *commission* errors were more prevalent in the copy condition compared to the pretest, post-test, and CTC groups, echoing the results of Experiment 1. This finding suggests that errorful testing and receiving corrective feedback may help reduce the occurrence of commission errors during subsequent recall, which contradicts previous hypotheses about the negative influence of errors. This aligns with research showing minimal intrusion errors in errorful learning conditions (Knight et al., 2012; Mera et al., 2025c). Additionally, the proportion of *confusion* errors was higher in the CTC and post-test group compared to the pretest and copy conditions. Therefore, administering the test before exposure to the material, as in the case of the pretest condition, may mitigate inter-list confusion errors. However, the absence of this effect in Experiment 1 suggests that further research is needed to clarify this relationship. Importantly, the rate of *intrusion* errors did not significantly differ between the pretest, post-test, and CTC conditions, which can be taken to indicate little interference from previous errors.

Participants in the pretest and copy conditions exhibited higher RTs than the post-test and CTC conditions. This finding is consistent with the results of Experiment 1 and suggests that conditions involving higher error rates (such as the pretest) may lead to slower RTs during subsequent recall. One potential explanation could be that pretesting enhances engagement, leading to more deliberate processing during retrieval, thus resulting in slower RTs. However, it is worth noting that these processing differences did not influence recall performance.

Finally, the analysis of metacognitive awareness revealed that participants tended to underestimate their performance on errorful learning conditions (pretest, post-test, and CTC), indicating a lack of awareness of the benefits of errorful testing for memory performance. Moreover, participants rated experiencing errors as less beneficial for learning than having correct answers, reflecting a metacognitive illusion that fluent study conditions (such as copying) are more effective for learning. This replicates the results of Experiment 1 and suggests that this metacognitive illusion may persist regardless of the additional encoding opportunity provided after errorful testing. In summary, Experiment 2 confirms several findings from Experiment 1 while providing further insights into the implications of test order and error generation for memory performance.

## Experiment 3

Another aim of the present study was to explore the generalizability of the pretesting effect across different age populations. While most studies on pretesting have focused on neurologically typical young adults, it remains unclear whether this effect extends to older populations. Experiment 3 aimed to investigate whether the pretesting effect persists in older adults, addressing a gap in our understanding of age-related learning strategies.

Previous studies on errorful learning in aging populations (Cyr & Anderson, 2012, 2015; Kessel & de Haan, 2003; Metcalfe et al., 2015) suggest that older adults can still benefit from errorful learning strategies such as pretesting. Accordingly, we expected that older adults in the pretest condition would outperform those in the copy condition, providing evidence that pretesting remains effective across different age groups. Additionally, given that older adults may be more susceptible to memory interference (Biss et al., 2013; Hasher & Zacks, 1988; Jacoby et al., 2001), we predicted that they would exhibit a higher proportion of intrusion errors compared to younger adults. Furthermore, in line with well-documented age-related declines in processing speed (e.g., Verhaeghen, 2016) and increased RTs (e.g., Rhodes et al., 2019), we expected older adults to take longer to respond than younger adults, yet still retain the benefits of pretesting. Finally, we anticipated that older adults, like their younger counterparts, would underestimate the efficacy of errorful learning.

The experimental procedure and materials were largely identical to Experiment 1, with one notable exception: the absence of a post-test group. This decision to focus exclusively on pretesting in older adults was made to allow for a more in-depth exploration of this specific effect in an understudied population. However, it is important to acknowledge that this design choice limits our ability to directly compare the efficacy of pretesting versus post-testing in older adults. Despite this limitation, Experiment 3 still offers crucial insights into the generalizability of the pretesting effect across age groups. By focusing specifically on older adults, this experiment aimed to shed light on how aging may influence the effectiveness of pretesting as a learning strategy.

### Method

#### Participants

The participants were 47 older adults recruited from University for Senior Citizens at the University of the Basque Country UPV/EHU. They were randomly allocated to two groups: The Pretest group (n = 24; 16 females, eight males; *M*_age_ = 66.79, *SD*_age_ = 4.21 years) and the Copy group (n = 23; 17 females, six males; *M*_age_ = 67.48, *SD*_age_ = 3.36 years). All participants were healthy older adults with no cognitive impairments. They completed a mini-mental state examination (Folstein et al., 1975; adapted to Spanish: Lobo et al., 1979) and scored 24 or higher (out of 30; *M* = 29.28, *SD* = 0.68). A processing speed test was also administered (Digit Symbol Substitution test, WAIS-IV; Wechsler, 2012), where participants were asked to copy a series of symbols paired with a number. The test required participants to draw each symbol under its corresponding number as quickly as possible. No statistically significant differences were found between the groups regarding performance on this test (scalar scores: pretest, *M* = 12.78, *SD* = 1.93 vs. read, *M* = 12.75, *SD* = 1.98, *p* >.05).

#### Design

The experiment adopted a between-subjects design with an independent variable, learning condition, which had two levels: pretest versus copy. The dependent variable was the number of correctly recalled target words when cued with their corresponding cue-words during the final test phase.

### Results

#### Cued-recall performance

**Fig. 5:**
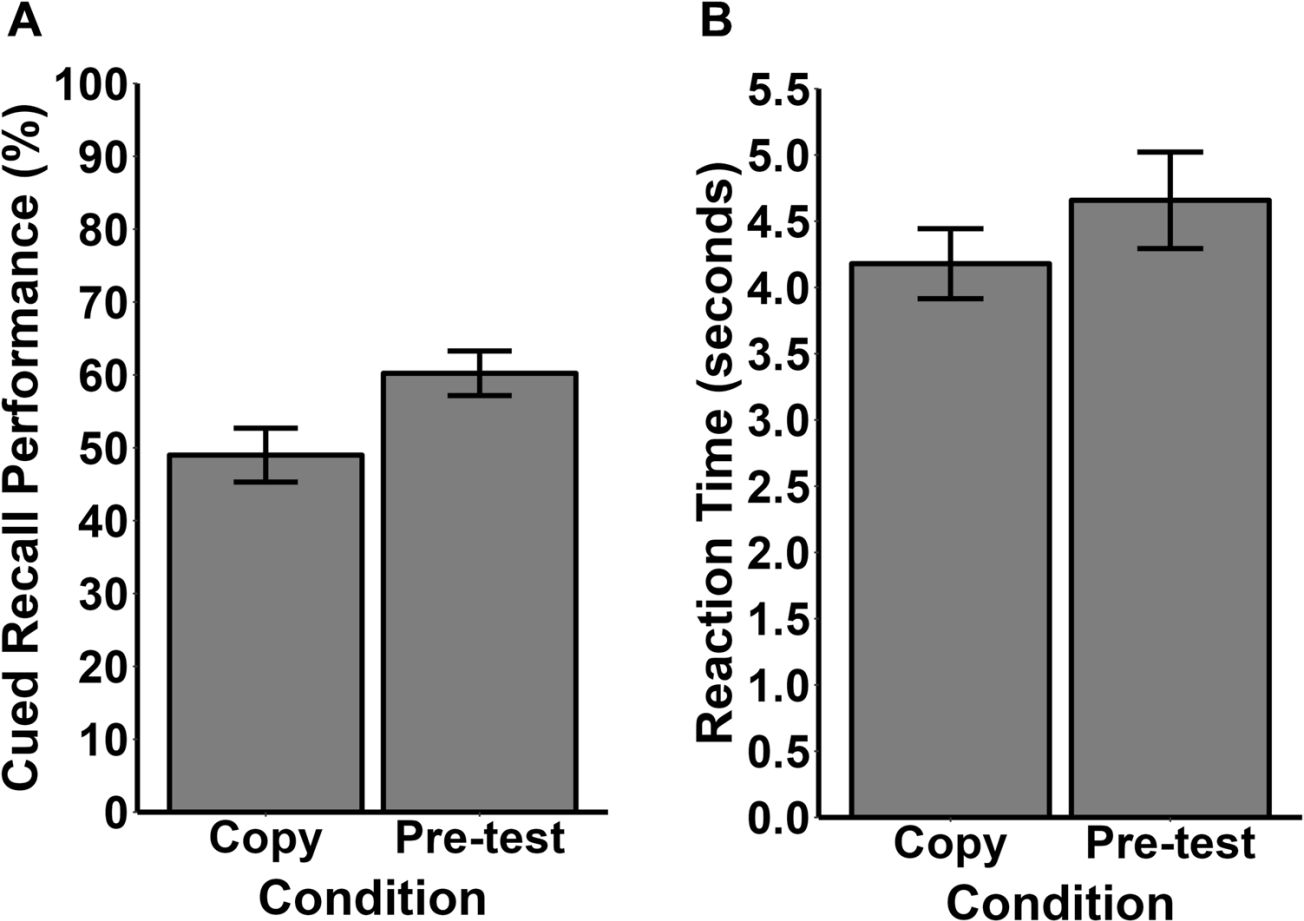
Results of Experiment 3 comparing Pretest and Copy groups. (**A**) Mean percentage of cued-recall test accuracy. (**B**) Reaction time (in seconds) at the final test

In the case of older adults, the pretest (*M* = 60.23, *SD* = 14.68) still yielded a higher cued-recall performance than the copy condition (*M* = 48.99, *SD* = 18.11), *t*(45) = 2.33, *p* =.02, *d* = 0.68 (see Fig. 5A). These results indicate that the pretesting effect extends to senior citizens.

#### Error-type analysis

We examined the types of errors made by participants in each learning condition (see Table 1). More commission errors were made in the copy (*M* =.85, *SD* =.08) than the pretest learning condition (*M* =.62, *SD* =.15), *t*(45) = 6.68, *p* <.001, *d* = 1.95. Regarding confusion errors, no significant differences were found between the pretest (*M* =.19, *SD* =.07) and copy conditions (*M* =.15, *SD* =.08), *t*(45) = 1.84, *p* =.07, *d* = 0.54. Finally, the proportion of intrusion errors accounted for only.20 (*SD* =.12) of the total errors made by the pretest group.

#### Reaction times

Participants in the pretest condition took slightly longer to respond (*M* = 4.67, *SD* = 1.75) than those in the copy condition (*M* = 4.18, *SD* = 1.29). However, this difference was not significant, *t*(45) = 1.07, *p* =.29, *d* = 0.31 (see Fig. 5B).

#### Metacognitive questions

Participants rated their performance lower than their actual performance in the pretest condition (44.63 vs. 60.23), *t*(44) = 2.98, *p* =.005, *d* = 0.88. In contrast, in the copy condition, rated and actual performance did not differ (46.71 vs. 48.99), *t*(46) = 0.41, *p* =.68, *d* = 0.12. Thus, participants underestimated their performance in the errorful pretest conditions. Additionally, no differences were found in the participants’ ratings concerning the perceived benefit of errors for their learning. Participants rated errors as beneficial for their learning similarly in both pretest (*M* = 42.26%, *SD* = 25.92) and copy conditions (*M* = 48.03%, *SD* = 23.79; *t*(46) = 0.88, *p* =.38, *d* = 0.26).

#### Cross-experimental comparison of older and younger adults

We also aimed to compare the results of the young participants from Experiment 1 with those of the older adults of Experiment 3. To achieve this, we conducted a between-experiment comparison of cued recall performance in the final test using a 2 (learning condition: pretest, copy) × 2 (age group: young, older adults) between-subjects ANOVA. This analysis revealed a significant main effect of learning condition on cued recall performance, *F*(1, 105) = 12.18, *p* =.001, ${\eta }_{g}^{2}$ =.10. As expected, there was a learning condition effect, with participants in the pretest group performing significantly better than those in the copy group. Notably, there was no significant main effect of age group, *F*(1, 105) = 1.1, *p* =.29, ${\eta }_{g}^{2}$ =.01, or an interaction between age group and learning condition, *F*(1, 105) = 0.02, *p* =.88, ${\eta }_{g}^{2}$ <.01. These results suggest that the beneficial effects of pretesting on memory performance are independent of age, at least for young and healthy older participants.

Further analysis of each error type revealed no significant effect of age group for commission errors, *F*(1, 105) = 1.13, *p* =.29, ${\eta }_{g}^{2}$ =.01, or an interaction between age group and learning condition, *F*(1, 105) = 0.12, *p* =.73, ${\eta }_{g}^{2}$ <.01. Similarly, there were no age-group effects for confusion errors, *F*(1, 105) = 0.36, *p* =.55, ${\eta }_{g}^{2}$ <.01, or an interaction between age group and learning condition, *F*(1, 105) = 2.10, *p* =.15, ${\eta }_{g}^{2}$ =.02. Additionally, no differences were found in the proportion of intrusion errors between young and older participants, *t*(52) = 0.96, *p* =.34, *d* = 0.26.

Regarding RTs, we observed a significant main effect of age group, *F*(1, 105) = 16.34, *p* <.001, ${\eta }_{g}^{2}$ =.13, and no interaction between learning condition and age group, *F*(1, 105) = 0.03, *p* =.86, ${\eta }_{g}^{2}$ <.01. Older participants took longer to respond than young participants in both the pretest condition (*M* = 4.67, *SD* = 1.75 vs. *M* = 3.64, *SD* = 0.79; *t*(52) = 2.89, *p* =.006, *d* = 0.80), and copy conditions (*M* = 4.18, *SD* = 1.29 vs. *M* = 3.24, *SD* = 1.17; *t*(53) = 2.82, *p* =.007, *d* = 0.77), with a Bonferroni correction of *p* =.025 (= 0.05/4).

Finally, regarding the two metacognitive measures, there was no significant effect of age group regarding the ratings given by participants when predicting their performance, *F*(1, 105) = 0.38, *p* =.54, ${\eta }_{g}^{2}$ <.01, or interaction between age group and learning condition, *F*(1, 105) = 0.49, *p* =.48, ${\eta }_{g}^{2}$ <.01. However, there was a statistically significant difference between age groups regarding the perceived effectiveness of correct answers versus errors in their learning, *F*(1, 105) = 6.75, *p* =.01, ${\eta }_{g}^{2}$ =.06, with no interaction between learning condition and age group, *F*(1, 105) = 1.46, *p* =.23, ${\eta }_{g}^{2}$ =.01. In the Copy condition, older participants rated errors as less beneficial for their learning (*M* = 48.03, *SD* = 23.79) compared to younger adults (*M* = 64.00, *SD* = 15.89; *t*(53) = 3.28, *p* =.002, *d* = 0.89). This difference was not significant in the pretest condition (*M* = 42.26, *SD* = 25.92 vs. *M* = 48.17, *SD* = 19.95; *t*(52) = 0.85, *p* =.40, *d* = 0.23).

In summary, the cross-experimental comparison revealed that the beneficial effects of pretesting on cued recall performance were consistent across the young and older adult groups despite certain age-related differences in RTs and metacognitive judgments.

### Discussion

Experiment 3 aimed to investigate whether the pretesting effect, well documented in a younger population, could also benefit older adults. Consistent with previous studies (e.g., Cyr & Anderson, 2015), older adults in the Pretest condition exhibited higher cued recall performance than those in the Copy condition. This finding suggests that the pretest effect extends to healthy older adults, indicating that errorful testing can also enhance memory performance in this population.

The extension of the pretesting effect to older adults holds particular significance in the context of age-related memory decline. Since cognitive abilities, especially episodic memory, often diminish with age (e.g., Nyberg et al., 2012), identifying effective strategies to support memory function in older adults is essential. Our findings suggest that pretesting may serve as a valuable tool for mitigating certain aspects of age-related memory decline, potentially helping older adults to maintain better cognitive function and enhance everyday recall.

Importantly, there were no significant age-group differences in cued-recall performance (young $\approx$ seniors), indicating that the pretest effect is robust across different age groups. However, we did not observe typical differences between young and older adults in episodic memory, as both age groups showed similar performance in cued recall. Previous research has indicated better performance in young adults than their older counterparts (Rhodes et al., 2019). The absence of such differences between young and older adults in our study may be attributed to a potential bias in sample selection since participants were enrolled in the University for Senior Citizens program and may be more cognitively active than other aging populations. This limitation highlights the need for future research to explore the pretesting effect in a more diverse sample of older adults, including those with varying levels of cognitive function and those experiencing more pronounced age-related memory decline.

Older adults in the copy condition made more commission errors compared to those in the pretest condition, indicating that errorful testing may help reduce the occurrence of commission errors during subsequent recall. This finding is particularly relevant for older adults who may be more susceptible to false memories or confabulations (e.g., Devitt & Schacter, 2016). By potentially reducing commission errors, pretesting could help older adults maintain more accurate memories. However, there were no significant differences in confusion errors between the two conditions, thus errorful testing may not influence the occurrence of confusion errors in older adults. Furthermore, the relatively low proportion of intrusion errors (20%) among older adults’ incorrect responses is noteworthy, especially given that prior research has shown increased memory interference with aging. This discrepancy warrants further exploration. One possible explanation is that our task, which used semantically related word pairs, might have facilitated the use of semantic strategies in older adults. Semantic strategies can sometimes compensate for age-related declines in other cognitive functions (e.g., Naveh-Benjamin, 2000), which could potentially reduce intrusion errors. Another factor could be the specific nature of the interference. While aging may increase susceptibility to interference from previously learned information (Biss et al., 2013; Hasher & Zacks, 1988; Jacoby et al., 2001), our study focused on interference within a relatively short learning interval. It is possible that the effects of aging on proactive interference are more pronounced over longer retention intervals. Future research should investigate these possibilities by manipulating the semantic relatedness of materials, varying retention intervals, and examining individual differences in cognitive abilities within older adult samples.

Like young adults, older adults in the pretest condition tended to underestimate their performance and were mostly unaware of the benefits of errorful testing for memory. In contrast, participants in the copy condition accurately assessed their performance, indicating a lack of metacognitive awareness regarding the benefits of errorful testing in older adults. This discrepancy highlights a potential metacognitive bias that could impair the effective use of pretesting in educational and cognitive intervention settings for older adults.

The cross-experimental comparison between young and older adults revealed that the beneficial effects of pretesting on final cued recall were consistent across age groups. Both young and older adults showed better recall in the pretest condition compared to the copy condition, with no age differences. Similarly, there were no significant age-group differences in the types of errors (commission, confusion, or intrusion) made by participants. This consistency indicates that the beneficial effect of pretesting may be preserved across the adult lifespan, at least in a cognitively active population.

Regarding RTs, consistent with previous studies (e.g., Rhodes et al., 2019), older adults exhibited longer RTs than young adults, indicating they required more time to respond during the cued recall task. This finding aligns with the well-established age-related decline in processing speed (e.g., Verhaeghen, 2016). However, the lack of interaction between age group and learning condition suggests that the benefits of pretesting are not significantly affected by age-related slowing, further supporting the potential of this strategy for older adults.

It is important to acknowledge that the sample size for the older adult group may be a limitation due to a post hoc power analysis (G*Power, Faul et al., 2007) that showed that our sample had a power of 0.74 (α =.05) to detect the effect size of 0.68 in the cued-recall *t*-test. Given that cognitive aging research often benefits from larger samples, this limited power might have influenced the results.

Our finding of preserved pretesting benefits in older adults, despite age-related changes in processing speed, presents a complex perspective when considered within broader theories of cognitive aging. For instance, the inhibitory deficit account posits that older adults have a reduced ability to inhibit irrelevant information (Hasher & Zacks, 1988), which often leads to increased interference and poorer memory performance (Biss et al., 2013; Ebert & Anderson, 2009; May et al., 1999). While we did observe increased RTs in older adults, consistent with this theory and general age-related slowing, the pretesting effect was not diminished, and errors did not substantially persist from the initial to the final test, nor were they more common compared to younger adults. This suggests that the benefits of pretesting might, in some way, circumvent or compensate for inhibitory deficits. Alternatively, the associative deficit hypothesis suggests that older adults experience specific difficulties in forming new associations (Naveh-Benjamin, 2000; Old & Naveh-Benjamin, 2008). The pretesting paradigm used in our study encourages the active creation of new associations between cues and targets. The fact that pretesting was beneficial implies that this associative deficit can be partially overcome through errorful testing, or that pretesting relies more on the strengthening of pre-existing semantic associations.

Overall, Experiment 3 provides compelling evidence that the pretest effect extends to older adults, suggesting that errorful testing can enhance memory performance in this population. These findings underscore the efficacy of pretesting as a learning strategy and its potential application across diverse populations. This study demonstrates the robustness of the pretesting effect across age groups, deepening our understanding of cognitive aging and offering promising strategies for supporting cognitive function in older adults. Further research is needed to fully understand the boundary conditions and mechanisms of the pretesting effect across the adult lifespan, particularly those experiencing cognitive decline. Additionally, future studies could examine the role of a broader range of baseline cognitive abilities, such as working memory, in pretesting effects, especially within older adult populations.

## General discussion

The present study explored the impact of test order on memory. Our findings from three experiments provide valuable insights into the pretest procedure as a relevant learning tool and its implications for memory encoding and retrieval processes.

Our results consistently demonstrate that engaging in testing during learning – whether as a pretest or post-test – enhances memory performance compared to errorless learning methods such as copying. Both pretesting and retrieval practice led to superior memory performance compared to copying, suggesting that engaging in active retrieval processes during learning is more beneficial than other, less cognitively active encoding processes, such as copying. Our results align closely with prior research, providing further support for the efficacy and robustness of errorful learning strategies such as pretesting and retrieval practice (e.g., Latimier et al., 2019; Pan & Sana, 2021). Our experiments employed a short retention interval (approximately 5 min), similar to Pan and Sana (2021), yielding consistent results. However, further research should investigate whether these findings between pretesting and retrieval practice hold over longer retention intervals, as observed by Latimier et al. (2019). Given that the retrieval practice effect is more evident with longer retention intervals (e.g., Dunlosky et al., 2013; Roediger & Karpicke, 2006b), the difference between the pretest and post-test may emerge when delaying the time between the initial and final test.

Moreover, our findings suggest that the pretesting effect extends to older adults over 60 years of age, indicating that errorful testing can also enhance memory performance in this age group. This is consistent with previous research demonstrating the benefits of trial-and-error learning among healthy older adults (Cyr & Anderson, 2015). Despite older adults showing slower processing speeds, there were no significant differences in pretest accuracy compared to younger adults, indicating the robust effectiveness of the pretesting method across different age groups. These insights can help to inform the development of effective interventions and educational strategies that promote lifelong learning in aging populations.

Furthermore, our analysis of error types revealed interesting insights into the nature of errors generated during learning. While commission errors were prevalent across all conditions, the occurrence of intrusion errors was minimal (< 26%), suggesting minimal proactive interference from previous errors during subsequent recall. This suggests that the act of testing itself may mitigate interference effects, contributing to better memory retention. Our findings are consistent with previous research (Knight et al., 2012; Mera et al., 2025a, 2025b, 2025c) investigating the impact of errorful learning conditions on intrusion errors. These studies have shown that commission errors were the most prevalent error type, while intrusion errors accounted for less than 10% of the incorrect responses made during the final test.

However, it is important to recognize that errorful learning conditions, particularly pretesting, were linked to longer RTs during the final recall task. This phenomenon can be attributed to the unique demands of pretesting, where participants engage in effortful retrieval only during the final cued-recall test. In contrast, post-testing and CTC conditions involve retrieval practice during both the learning phase and the final test, potentially enhancing fluency and reducing RTs. These results are also consistent with prior research by Huelser and Metcalfe (2012), which found that participants were slower to respond to items for which they had previously generated an error. This phenomenon, known as *post-error slowing*, indicates that committing errors slows down subsequent retrieval (Rabbitt & Rodgers, 1977). Post-error slowing is typically viewed as enhancing attention following errors (see Dutilh et al., 2012, for a review). However, in our experiments, slower RTs during pretesting did not translate into improved performance.

Integrating Pan and Carpenter’s (2023) three-stage framework of pretesting effects provides further insight into these RT differences. According to their model, pretesting influences learning through three stages: (1) initial retrieval attempts during pretesting, (2) changes in subsequent learning behaviors due to these attempts, and (3) retrieval processes during the final test. In our study, participants in the pretesting condition engaged in retrieval attempts during the initial pretest, which likely influenced their learning behaviors thereafter. This suggests that the timing and nature of retrieval practice can differentially impact the efficiency of retrieval processes during final assessments.​ Additionally, it is important to note that the final test response was not time-limited in our experimental procedure, unlike real-life scenarios such as exams. Future research should examine whether differences between pretest and post-test conditions emerge when responses are time-limited.

Another significant aspect of our study is the discrepancy between participants’ perceived and actual performance. Participants tended to underestimate the benefits of errorful testing for memory performance, indicating a lack of metacognitive awareness. When asked to judge the effectiveness of experiencing errors versus correct answers during learning, participants rated having correct answers as more beneficial for their learning. This preference for fluent processing aligns with previous research suggesting that learners often perceive conditions that facilitate smooth and effortless learning, such as copying, as more effective than those involving effort (Bjork & Bjork, 2011). This pattern has been observed in studies on the pretesting effect (Huelser & Metcalfe, 2012; Yang et al., 2017) and the retrieval practice effect (Kornell & Son, 2009). However, it is important to note that conditions presenting challenges and effort during learning often lead to enhanced long-term retention, referred to as “desirable difficulties” (Bjork & Bjork, 2011). Interestingly, recent research suggests that this lack of awareness of the pretesting benefit could potentially be mitigated through self-regulation strategies (Pan & Rivers, 2023). Overall, our findings underscore the common metacognitive illusion that conditions favoring fluent learning are perceived as superior despite not necessarily being objectively more effective.

The present study’s findings align closely with prior research, providing further support for the efficacy and robustness of errorful learning strategies such as pretesting and retrieval practice (e.g., Latimier et al., 2019; Pan & Sana, 2021). However, it is crucial to consider the boundary conditions under which these strategies are more effective. Pretesting effects have been demonstrated across various types of learning materials, including word pairs (Clark, 2016; Grimaldi & Karpicke, 2012; Vaughn & Rawson, 2012), factual information (Kang et al., 2011; Richland et al., 2009), sentence translations (Guzmán-Muñoz, 2020), and video lectures (Toftness et al., 2018), in both laboratory and classroom settings (e.g., Carpenter et al., 2018), and using both cued-recall and multiple-choice formats (e.g., Little & Bjork, 2016).

Similarly, retrieval practice has been shown to enhance learning across a wide array of materials (Duchon et al., 2013), including word pair lists (e.g., Karpicke & Roediger, 2008; Roediger & Karpicke, 2006a), educational texts (Agarwal et al., 2021; Carpenter et al., 2009; McDaniel et al., 2007), video lectures (e.g., Butler & Roediger, 2007), using both free-recall (e.g., Roediger & Karpicke, 2006b) and cued-recall formats (e.g., Vaughn & Rawson, 2011). These findings indicate that both pretesting and retrieval practice can produce robust learning benefits across different content types, learning environments, and assessment formats. Future research should explore whether the relative effectiveness of pretesting and retrieval practice is moderated by factors such as material type and learner characteristics, and how these findings generalize to other learning domains beyond word pairs, such as conceptual learning or problem-solving tasks.

### Future directions

Although this study has addressed several key questions about errorful learning, several research questions remain open. Future research should refine our understanding of pretesting’s mechanisms and its applicability across diverse populations, materials, and conditions. Specifically, follow-up studies should examine the impact of varying retention intervals between learning and testing, and the influence of material complexity by moving beyond word pairs to more ecologically valid materials like conceptual problem-solving tasks or multimedia content. Individual differences in errorful learning also warrant further study. In that sense, characteristics such as motivation, working memory capacity, and metacognitive skills should be considered as possible modulatory factors. Additionally, we have shown the efficacy of pretesting effect in an older population, but it is important to extend research to special populations – such as individuals with cognitive impairments or neurodevelopmental conditions – which could help clarify the boundaries of errorful learning’s applicability (e.g., Clare & Jones, 2008).

### Educational implications

The present study offers several important implications for educational practice. The consistent benefit of incorporating tests during learning, compared to passive copying, highlights the need to incorporate active retrieval strategies into teaching methodologies. Although pretesting may seem counterintuitive, given that it involves testing students on material they have not yet learned, our findings suggest it can be a powerful tool to enhance subsequent learning. Educators should consider implementing low-stakes pretests or quizzes at the beginning of a learning unit to activate prior knowledge, be aware of their intuitive responses, and prepare students to encode new and correct information more effectively. In addition, the CTC condition, with its repeated study-test cycles approach, encourages students to actively engage with the material, identify gaps in their knowledge, and revisit the material, potentially leading to deeper learning and retention.

It is important to acknowledge that incorporating tests can raise concerns about the potential reinforcement of errors, especially during difficult tests. Such concerns may discourage educators from adopting errorful learning approaches. However, in our study, participants did not exhibit a significant increase in intrusion errors during the final tests, indicating that errorful learning does not promote substantial proactive interference.

Finally, the observed metacognitive unawareness of the benefits of errorful learning highlights the importance of educating teachers and students about effective learning strategies. Students may intuitively believe that passive copying is more effective, but educators should explicitly teach them about the power of active retrieval and the value of making errors as part of the learning process. Addressing this metacognitive bias against errorful learning strategies could be key to improving learning outcomes (Pan & Rivers, 2023).

Overall, this research provides further evidence that incorporating testing into educational practices, even when it involves errors, can significantly improve learning outcomes. By understanding the mechanisms of errorful learning and addressing metacognitive misperceptions, educators can create more effective and engaging learning environments.

### Conclusion

In conclusion, the present study significantly enhances our understanding of the nature of errorful learning and its impact on memory across different age groups. Our findings provide robust evidence for the beneficial impact of testing during learning on enhancing memory. Both pretesting and retrieval practice led to superior memory performance compared to errorless learning methods such as copying. While both pretesting and retrieval practice enhance memory, our results indicate potential differences in their underlying mechanisms. Notably, the pretesting conditions were associated with longer RTs during the final cued-recall test, suggesting that pretesting may involve a more effortful or different retrieval process. Overall, our findings emphasize the importance of incorporating errorful learning strategies into educational practices to enhance memory performance and promote effective learning across the lifespan. Future research should delve deeper into the specific cognitive processes underlying errorful learning and develop tailored interventions for optimizing learning outcomes in diverse populations.

## Declarations

### Data, material, and code availability

All data, material, and code from this study are available on the Open Science Framework (https://osf.io/48yua/).

### Open Practices Statement

All data, analytic code, and materials needed to replicate this study are available on the Open Science Framework (https://osf.io/48yua/). None of the reported experiments was preregistered.

## References

[CR1] Agarwal, P. K., Nunes, L. D., & Blunt, J. R. (2021). Retrieval practice consistently benefits student learning: A systematic review of applied research in schools and classrooms. *Educational Psychology Review,**33*(4), 1409–1453. 10.1007/s10648-021-09595-9

[CR2] Arnold, K. M., & McDermott, K. B. (2013). Test-potentiated learning: Distinguishing between direct and indirect effects of tests. *Journal of Experimental Psychology: Learning Memory and Cognition, 39*(3), 940–945. 10.1037/a0029199 10.1037/a0029199 PMC3764602 22774852

[CR3] Baddeley, A., & Wilson, B. A. (1994). When implicit learning fails: Amnesia and the problem of error elimination. *Neuropsychologia,**32*(1), 53–68. 10.1016/0028-3932(94)90068-X 8818154 10.1016/0028-3932(94)90068-x

[CR4] Balota, D. A., Duchek, J. M., Sergent-Marshall, S. D., & Roediger, H. L., 3rd. (2006). Does expanded retrieval produce benefits over equal-interval spacing? Explorations of spacing effects in healthy aging and early stage Alzheimer’s disease. *Psychology and Aging,**21*(1), 19–31. 10.1037/0882-7974.21.1.19 16594788 10.1037/0882-7974.21.1.19

[CR5] Bandura, A. (1986). *Social foundations of thought and action: A social cognitive theory*. Prentice-Hall.

[CR6] Biss, R. K., Campbell, K. L., & Hasher, L. (2013). Interference from previous distraction disrupts older adults’ memory. *The Journals of Gerontology. Series b, Psychological Sciences and Social Sciences,**68*(4), 558–561. 10.1093/geronb/gbs074 22929391 10.1093/geronb/gbs074

[CR7] Bjork, E. L., & Bjork, R. A. (2011). Making things hard on yourself, but in a good way: Creating desirable difficulties to enhance learning. In M. A. Gernsbacher, R. W. Pew, L. M. Hough, & J. R. Pomerantz (Eds.), *Psychology and the real world: Essays illustrating fundamental contributions to society* (pp. 56–64). Worth Publishers.

[CR8] Bridges, D., Pitiot, A., MacAskill, M. R., & Peirce, J. W. (2020). The timing mega-study: Comparing a range of experiment generators, both lab-based and online. *PeerJ,**8*, e9414. 10.7717/peerj.9414 33005482 10.7717/peerj.9414 PMC7512138

[CR9] Butler, A. C., & Roediger, H. L., III. (2007). Testing improves long-term retention in a simulated classroom setting. *European Journal of Cognitive Psychology,**19*(4–5), 514–527. 10.1080/09541440701326097

[CR10] Butterfield, B., & Metcalfe, J. (2001). Errors committed with high confidence are hypercorrected. *Journal of Experimental Psychology: Learning, Memory, and Cognition,**27*(6), 1491–1494. 10.1037/0278-7393.27.6.1491 11713883 10.1037//0278-7393.27.6.1491

[CR11] Carpenter, S. K. (2009). Cue strength as a moderator of the testing effect: The benefits of elaborative retrieval. *Journal of Experimental Psychology: Learning, Memory, and Cognition,**35*(6), 1563–1569. 10.1037/a0017021 19857026 10.1037/a0017021

[CR12] Carpenter, S. K. (2011). Semantic information activated during retrieval contributes to later retention: Support for the mediator effectiveness hypothesis of the testing effect. *Journal of Experimental Psychology: Learning, Memory, and Cognition,**37*(6), 1547–1552. 10.1037/a0024140 21707217 10.1037/a0024140

[CR13] Carpenter, S. K., Pashler, H., & Cepeda, N. J. (2009). Using tests to enhance 8th grade students’ retention of U.S. history facts. *Applied Cognitive Psychology,**23*(6), 760–771. 10.1002/acp.1507

[CR14] Carpenter, S. K., Rahman, S., & Perkins, K. (2018). The effects of prequestions on classroom learning. *Journal of Experimental Psychology-Applied,**24*(1), 34–42. 10.1037/xap0000145 29595303 10.1037/xap0000145

[CR15] Chan, J. C. K., Meissner, C. A., & Davis, S. D. (2018). Retrieval potentiates new learning: A theoretical and meta-analytic review. *Psychological Bulletin, 144*(11), 1111–1146. 10.1037/bul0000166 10.1037/bul0000166 30265011

[CR16] Clare, L., & Jones, R. S. P. (2008). Errorless learning in the rehabilitation of memory impairment: A critical review. *Neuropsychology Review,**18*(1), 1–23. 10.1007/s11065-008-9051-4 18247118 10.1007/s11065-008-9051-4

[CR17] Clark, C. M. (2016). *When and Why does Learning Profit from the Introduction of Errors?* (Doctoral Dissertation, University of California Los Angeles). Retrieved from https://escholarship.org/uc/item/6zv5867p

[CR18] Collins, A. M., & Loftus, E. F. (1975). A spreading-activation theory of semantic processing. *Psychological Review,**82*(6), 407–428. 10.1037/0033-295X.82.6.407

[CR19] Craik, F. I. M., & Tulving, E. (1975). Depth of processing and the retention of words in episodic memory. *Journal of Experimental Psychology: General,**104*(3), 268–294. 10.1037/0096-3445.104.3.268

[CR20] Cyr, A. A., & Anderson, N. D. (2012). Trial-and-error learning improves source memory among young and older adults. *Psychology and Aging,**27*(2), 429–439. 10.1037/a0025115 21859216 10.1037/a0025115

[CR21] Cyr, A. A., & Anderson, N. D. (2015). Mistakes as stepping stones: Effects of errors on episodic memory among younger and older adults. *Journal of Experimental Psychology: Learning, Memory, and Cognition,**41*(3), 841–850. 10.1037/xlm0000073 25347615 10.1037/xlm0000073

[CR22] Cyr, A. A., & Anderson, N. D. (2018). Learning from your mistakes: Does it matter if you’re out in left foot, I mean field? *Memory,**26*(9), 1281–1290. 10.1080/09658211.2018.1464189 29659332 10.1080/09658211.2018.1464189

[CR23] Devitt, A. L., & Schacter, D. L. (2016). False memories with age: Neural and cognitive underpinnings. *Neuropsychologia,**91*, 346–359. 10.1016/J.NEUROPSYCHOLOGIA.2016.08.030 27592332 10.1016/j.neuropsychologia.2016.08.030 PMC5075259

[CR24] DiMarco, D., Laursen, S. J., Churey, K. R., & Fiacconi, C. M. (2024). Examining the influence of list composition on the mnemonic benefit of errorful generation. *Memory,**33*(2), 145–156. 10.1080/09658211.2024.2413159 39401318 10.1080/09658211.2024.2413159

[CR25] Duchon, A., Perea, M., Sebastián-Gallés, N., Martí, A., & Carreiras, M. (2013). Espal: One-stop shopping for Spanish word properties. *Behavior Research Methods,**45*(4), 1246–1258. 10.3758/s13428-013-0326-1 23468181 10.3758/s13428-013-0326-1

[CR26] Dunlosky, J., Rawson, K. A., Marsh, E. J., Nathan, M. J., & Willingham, D. T. (2013). Improving students’ learning with effective learning techniques: Promising directions from cognitive and educational psychology. *Psychological Science in the Public Interest,**14*(1), 4–58. 10.1177/1529100612453266 26173288 10.1177/1529100612453266

[CR27] Dutilh, G., Vandekerckhove, J., Forstmann, B. U., Keuleers, E., Brysbaert, M., & Wagenmakers, E. J. (2012). Testing theories of post-error slowing. *Attention, Perception, & Psychophysics,**74*(2), 454–465. 10.3758/s13414-011-0243-2 10.3758/s13414-011-0243-2 PMC3283767 22105857

[CR28] Ebert, P. L., & Anderson, N. D. (2009). Proactive and retroactive interference in young adults, healthy older adults, and older adults with amnestic mild cognitive impairment. *Journal of the International Neuropsychological Society,**15*(1), 83–93. 10.1017/S1355617708090115 19128531 10.1017/S1355617708090115

[CR29] Faul, F., Erdfelder, E., Lang, A. G., & Buchner, A. (2007). G*Power 3: A flexible statistical power analysis program for the social, behavioral, and biomedical sciences. *Behavior Research Methods, 39*(2), 175–191. 10.3758/BF03193146 10.3758/bf03193146 17695343

[CR30] Fernández, A., Díez, E., & Alonso, M. A. (2010). Normas de Asociación libre en Castellano de la Universidad de Salamanca [Online database]. Retrieved from https://iblues-inico.usal.es/iblues/nalc_home.php

[CR31] Flavell, J. H., & Wellman, H. M. (1977). Metamemory. In R. V. Kail, & J. W. Hagen (Eds.), *Perspectives on the development of memory and cognition*. Lawrence Erlbau.

[CR32] Folstein, M. F., Folstein, S. E., & McHugh, P. R. (1975). “Mini-mental state”. *Journal of Psychiatric Research, 12*(3), 189–198. 10.1016/0022-3956(75)90026-6 10.1016/0022-3956(75)90026-6 1202204

[CR33] Grimaldi, P. J., & Karpicke, J. D. (2012). When and why do retrieval attempts enhance subsequent encoding? *Memory & Cognition,**40*(4), 505–513. 10.3758/s13421-011-0174-0 22238214 10.3758/s13421-011-0174-0

[CR34] Guzmán-Muñoz, F. J. (2020). Effects of making errors in learning a foreign language. *Journal of Cognitive Psychology,**32*(2), 229–241. 10.1080/20445911.2020.1711766

[CR35] Halamish, V., & Bjork, R. A. (2011). When does testing enhance retention? A distribution-based interpretation of retrieval as a memory modifier. *Journal of Experimental Psychology: Learning, Memory, and Cognition,**37*(4), 801–812. 10.1037/a0023219 21480751 10.1037/a0023219

[CR36] Hartley, J. (1973). The effect of pre-testing on post-test performance. *Instructional Science,**2*(2), 193–214. 10.1007/BF00139871

[CR37] Hasher, L., & Zacks, R. T. (1988). Working Memory, Comprehension, and Aging: A Review and a New View. *Psychology of Learning and Motivation,**22*, 193–225. 10.1016/S0079-7421(08)60041-9

[CR38] Hollins, T. J., Seabrooke, T., Inkster, A., Wills, A., & Mitchell, C. J. (2023). Pre-testing effects are target-specific and are not driven by a generalised state of curiosity. *Memory,**31*(2), 282–296. 10.1080/09658211.2022.2153141 36475537 10.1080/09658211.2022.2153141

[CR39] Huelser, B. J., & Metcalfe, J. (2012). Making related errors facilitates learning, but learners do not know it. *Memory & Cognition,**40*(4), 514–527. 10.3758/s13421-011-0167-z 22161209 10.3758/s13421-011-0167-z

[CR40] Izawa, C. (1970). Optimal potentiating effects and forgetting-prevention effects of tests in paired-associate learning. *Journal of Experimental Psychology,**83*, 340–344. 10.1037/h0028541

[CR41] Jacoby, L. L., Debner, J. A., & Hay, J. F. (2001). Proactive interference, accessibility bias, and process dissociations: Valid subjective reports of memory. *Journal of Experimental Psychology: Learning, Memory, and Cognition,**27*(3), 686–700. 10.1037/0278-7393.27.3.686 11394674

[CR42] Kang, S. H., McDermott, K. B., & Roediger, I. I. I., H. L. (2007). Test format and corrective feedback modify the effect of testing on long-term retention. *European Journal of Cognitive Psychology,**19*(4–5), 528–558. 10.1080/09541440601056620

[CR43] Kang, S. H. K., Pashler, H., Cepeda, N. J., Rohrer, D., Carpenter, S. K., & Mozer, M. C. (2011). Does incorrect guessing impair fact learning? *Journal of Educational Psychology,**103*(1), 48–59. 10.1037/a0021977

[CR44] Karpicke, J. D., & Aue, W. R. (2015). The testing effect is alive and well with complex materials. *Educational Psychology Review,**27*(2), 317–326. 10.1007/s10648-015-9309-3

[CR45] Karpicke, J. D., & Roediger, H. L. (2008). The critical importance of retrieval for learning. *Science,**319*(5865), 966–968. 10.1126/science.1152408 18276894 10.1126/science.1152408

[CR46] Kessels, R. P., & de Haan, E. H. (2003). Mnemonic strategies in older people: A comparison of errorless and errorful learning. *Age and Ageing,**32*(5), 529–533. 10.1093/ageing/afg068 12958003 10.1093/ageing/afg068

[CR47] Kliegl, O., Bartl, J., & Bäuml, K. H. T. (2023). The pretesting effect thrives in the presence of competing information. *Memory, 31*(5), 705–714. 10.1080/09658211.2023.2190568 10.1080/09658211.2023.2190568 36927213

[CR48] Knight, J. B., Hunter Ball, B., Brewer, G. A., DeWitt, M. R., & Marsh, R. L. (2012). Testing unsuccessfully: A specification of the underlying mechanisms supporting its influence on retention. *Journal of Memory and Language,**66*(4), 731–746. 10.1016/j.jml.2011.12.008

[CR49] Kornell, N. (2014). Attempting to answer a meaningful question enhances subsequent learning even when feedback is delayed. *Journal of Experimental Psychology: Learning, Memory, and Cognition,**40*(1), 106–114. 10.1037/a0033699 23855547 10.1037/a0033699

[CR50] Kornell, N., Hays, M. J., & Bjork, R. A. (2009). Unsuccessful retrieval attempts enhance subsequent learning. *Journal of Experimental Psychology: Learning, Memory, and Cognition,**35*(4), 989–998. 10.1037/a0015729 19586265 10.1037/a0015729

[CR51] Kornell, N., & Son, L. K. (2009). Learners’ choices and beliefs about self-testing. *Memory,**17*, 493–501. 10.1080/09658210902832915 19468957 10.1080/09658210902832915

[CR52] Latimier, A., Riegert, A., Peyre, H., Ly, S. T., Casati, R., & Ramus, F. (2019). Does pre-testing promote better retention than post-testing? *NPJ Science of Learning,**4*(1), 1–7. 10.1038/s41539-019-0053-1 31583117 10.1038/s41539-019-0053-1 PMC6760123

[CR53] Little, J. L., Bjork, E. L., Bjork, R. A., & Angello, G. (2012). Multiple-choice tests exonerated, at least of some charges: Fostering test-induced learning and avoiding test-induced forgetting. *Psychological Science,**23*(11), 1337–1344. 10.1177/0956797612443370 23034566 10.1177/0956797612443370

[CR54] Little, J. L., & Bjork, E. L. (2016). Multiple-choice pretesting potentiates learning of related information. *Memory and Cognition,**44*(7), 1085–1101. 10.3758/s13421-016-0621-z 27177505 10.3758/s13421-016-0621-z

[CR55] Lobo, A., Ezquerra, J., Gómez Burgada, F., Sala, J. M., & Seva Díaz, A. (1979). El miniexamen, cognoscitivo (un "test" sencillo, práctico, para detectar alteraciones intelectuales en pacientes médicos) [Cognocitive mini-test (a simple practical test to detect intellectual changes in medical patients)]. *Actas luso-espanolas de neurologia, psiquiatria y ciencias afines, 7*(3), 189–202. 474231

[CR56] Park, D. C., & Festini, S. B. (2017). Theories of memory and aging: A look at the past and a glimpse of the future. *The Journals of Gerontology. Series b, Psychological Sciences and Social Sciences,**72*(1), 82–90. 10.1093/geronb/gbw066 27257229 10.1093/geronb/gbw066 PMC5156492

[CR57] Pastötter, B., & Bäuml, K.-H.T. (2019). Testing enhances subsequent learning in older adults. *Psychology and Aging,**34*(2), 242–250. 10.1037/pag0000307 30335442 10.1037/pag0000307

[CR58] Potts, R., & Shanks, D. R. (2012). Can testing immunize memories against interference? *Journal of Experimental Psychology: Learning, Memory, and Cognition,**38*(6), 1780–1785. 10.1037/a0028218 22686838 10.1037/a0028218

[CR59] Maraver, M. J., Lapa, A., Garcia-Marques, L., Carneiro, P., & Raposo, A. (2022). Can we learn from errors? Retrieval facilitates the correction of false memories for pragmatic inferences. *PLoS ONE,**17*(8), e0272427. 10.1371/JOURNAL.PONE.0272427 35917361 10.1371/journal.pone.0272427 PMC9345471

[CR60] Marin-Garcia, E., Mattfeld, A. T., & Gabrieli, J. D. E. (2021). Neural correlates of long-term memory enhancement following retrieval practice. *Frontiers in Human Neuroscience,**15*, 584560. 10.3389/fnhum.2021.584560 33613206 10.3389/fnhum.2021.584560 PMC7889502

[CR61] May, C. P., Hasher, L., & Kane, M. J. (1999). The role of interference in memory span. *Memory & Cognition,**27*(5), 759–767. 10.3758/bf03198529 10540805 10.3758/bf03198529

[CR62] McDaniel, M. A., Anderson, J. L., Derbish, M. H., & Morrisette, N. (2007). Testing the testing effect in the classroom. *European Journal of Cognitive Psychology,**19*(4–5), 494–513. 10.1080/09541440701326154

[CR63] Melton, A. W., & Irwin, J. M. (1940). The influence of degree of interpolated learning on retroactive inhibition and the overt transfer of specific responses. *The American Journal of Psychology,**53*(2), 173–203. 10.2307/1417415 3322059

[CR64] Mera, Y., Dianova, N., & Marin-Garcia, E. (2025a). The pretesting effect exploring the impact of feedback and final test timing. *Journal of Cognition,**8*(1), 41. 10.5334/joc.455 40718859 10.5334/joc.455 PMC12292081

[CR65] Mera, Y., Migueles, M., & Marin-Garcia, E. (2025). Enhancing memory through error correction. *Psicologica, 46*(1), e17154 10.20350/DIGITALCSIC/17154

[CR66] Mera, Y., Modirrousta-Galian, A., Thomas, G., Higham, P. A., & Seabrooke, T. (2025c). Erring on the side of caution: Two failures to replicate the derring effect. *Journal of Experimental Psychology: General,**154*(3), 658–671. 10.1037/xge0001707 39804380 10.1037/xge0001707

[CR67] Mera, Y., Rodríguez, G., & Marin-Garcia, E. (2022). Unraveling the benefits of experiencing errors during learning: Definition, modulating factors, and explanatory theories. *Psychonomic Bulletin & Review,**29*, 753–765. 10.3758/S13423-021-02022-8 34820785 10.3758/s13423-021-02022-8

[CR68] Metcalfe, J. (2017). Learning from errors. *Annual Review of Psychology,**68*(1), 465–489. 10.1146/annurev-psych-010416-044022 27648988 10.1146/annurev-psych-010416-044022

[CR69] Metcalfe, J., Casal-Roscum, L., Radin, A., & Friedman, D. (2015). On teaching old dogs new tricks. *Psychological Science,**26*(12), 1833–1842. 10.1177/0956797615597912 26494598 10.1177/0956797615597912 PMC4679660

[CR70] Metcalfe, J., & Huelser, B. J. (2020). Learning from errors is attributable to episodic recollection rather than semantic mediation. *Neuropsychologia*. 10.1016/j.neuropsychologia.2019.107296 31811845 10.1016/j.neuropsychologia.2019.107296

[CR71] Metcalfe, J., & Miele, D. B. (2014). Hypercorrection of high confidence errors: Prior testing both enhances delayed performance and blocks the return of the errors. *Journal of Applied Research in Memory and Cognition,**3*(3), 189–197. 10.1016/j.jarmac.2014.04.001

[CR72] Metcalfe, J., & Shimamura, A. P. (Eds.). (1994). *Metacognition: Knowing about knowing*. The MIT Press. 10.7551/mitpress/4561.001.0001

[CR73] Meyer, A. N., & Logan, J. M. (2013). Taking the testing effect beyond the college freshman: Benefits for lifelong learning. *Psychology and Aging,**28*(1), 142–147. 10.1037/a0030890 23437897 10.1037/a0030890

[CR74] Middleton, E. L., & Schwartz, M. F. (2012). Errorless learning in cognitive rehabilitation: A critical review. *Neuropsychological Rehabilitation,**22*(2), 138–168. 10.1080/09602011.2011.639619 22247957 10.1080/09602011.2011.639619 PMC3381647

[CR75] Montoro-Membila, N., Maraver, M. J., Marful, A., & Bajo, T. (2025). How do older adults correct memory errors? The effects of practice and metacognitive strategies. *Aging, Neuropsychology, and Cognition, 32*(5), 659–689. https://doi.org.ehu.idm.oclc.org/10.1080/13825585.2025.2464583 10.1080/13825585.2025.2464583 39962714

[CR76] Naveh-Benjamin, M. (2000). Adult age differences in memory performance: Tests of an associative deficit hypothesis. *Journal of Experimental Psychology: Learning, Memory, and Cognition,**26*(5), 1170–1187. 10.1037/0278-7393.26.5.1170 11009251 10.1037//0278-7393.26.5.1170

[CR77] Notebaert, W., Houtman, F., Opstal, F. V., Gevers, W., Fias, W., & Verguts, T. (2009). Post-error slowing: An orienting account. *Cognition,**111*(2), 275–279. 10.1016/j.cognition.2009.02.002 19285310 10.1016/j.cognition.2009.02.002

[CR78] Nyberg, L., Lövdén, M., Riklund, K., Lindenberger, U., & Bäckman, L. (2012). Memory aging and brain maintenance. *Trends in Cognitive Sciences,**16*(5), 292–305. 10.1016/j.tics.2012.04.005 22542563 10.1016/j.tics.2012.04.005

[CR79] Old, S. R., & Naveh-Benjamin, M. (2008). Memory for people and their actions: Further evidence for an age-related associative deficit. *Psychology and Aging,**23*(2), 467–472. 10.1037/0882-7974.23.2.467 18573021 10.1037/0882-7974.23.2.467

[CR80] Pan, S. C., & Rivers, M. L. (2023). Metacognitive awareness of the pretesting effect improves with self-regulation support. *Memory & Cognition,**51*, 1461–1480. 10.3758/s13421-022-01392-1 36637644 10.3758/s13421-022-01392-1 PMC9839203

[CR81] Pan, S. C., & Sana, F. (2021). Pretesting versus posttesting: Comparing the pedagogical benefits of errorful generation and retrieval practice. *Journal of Experimental Psychology: Applied,**27*(2), 237–257. 10.1037/xap0000345 33793291 10.1037/xap0000345

[CR82] Peirce, J., Gray, J. R., Simpson, S., MacAskill, M., Höchenberger, R., Sogo, H., Kastman, E., & Lindeløv, J. K. (2019). PsychoPy2: Experiments in behavior made easy. *Behavior Research Methods,**51*(1), 195–203. 10.3758/s13428-018-01193-y 30734206 10.3758/s13428-018-01193-y PMC6420413

[CR83] Postman, L., & Underwood, B. J. (1973). Critical issues in interference theory. *Memory & Cognition,**1*, 19–40. 10.3758/BF03198064 24214472 10.3758/BF03198064

[CR84] Potts, R., & Shanks, D. R. (2014). The benefit of generating errors during learning. *Journal of Experimental Psychology: General,**143*(2), 644–667. 10.1037/a0033194 23815457 10.1037/a0033194

[CR85] Pyc, M. A., & Rawson, K. A. (2010). Why testing improves memory: Mediator effectiveness hypothesis. *Science,**330*(6002), 335. 10.1126/science.1191465 20947756 10.1126/science.1191465

[CR86] R Core Team. (2023). R: A language and environment for statistical computing. R Foundation for Statistical Computing, Vienna, Austria. https://www.r-project.org/

[CR87] Rabbitt, P., & Rodgers, B. (1977). What does a man do after he makes an error? An analysis of response programming. *Quarterly Journal of Experimental Psychology,**29*(4), 727–743. 10.1080/14640747708400645

[CR88] Rescorla, R. A., & Wagner, A. R. (1972). A theory of Pavlovian conditioning: Variations in the effectiveness of reinforcement and nonreinforcement. In A. H. Black & W. F. Prokasy (Eds.), *Classical conditioning II: Current research and theory* (pp. 64–99). Appleton-Century-Crofts.

[CR89] Rhodes, S., Greene, N. R., & Naveh-Benjamin, M. (2019). Age-related differences in recall and recognition: A meta-analysis. *Psychonomic Bulletin & Review,**26*(5), 1529–1547. 10.3758/s13423-019-01649-y 31396816 10.3758/s13423-019-01649-y

[CR90] Richland, L. E., Kornell, N., & Kao, L. S. (2009). The pretesting effect: Do unsuccessful retrieval attempts enhance learning? *Journal of Experimental Psychology: Applied,**15*(3), 243–257. 10.1037/a0016496 19751074 10.1037/a0016496

[CR91] Roediger, H. L., & Butler, A. C. (2011). The critical role of retrieval practice in long-term retention. *Trends in Cognitive Sciences,**15*(1), 20–27. 10.1016/j.tics.2010.09.003 20951630 10.1016/j.tics.2010.09.003

[CR92] Roediger, H. L., & Karpicke, J. D. (2006a). The power of testing memory: Basic research and implications for educational practice. *Perspectives on Psychological Science,**1*(3), 181–210. 10.1111/j.1745-6916.2006.00012.x 26151629 10.1111/j.1745-6916.2006.00012.x

[CR93] Roediger, H. L., & Karpicke, J. D. (2006b). Test-enhanced learning: Taking memory tests improves long-term retention. *Psychological Science,**17*(3), 249–255. 10.1111/j.1467-9280.2006.01693.x 16507066 10.1111/j.1467-9280.2006.01693.x

[CR94] Rowland, C. A. (2014). The effect of testing versus restudy on retention: A meta-analytic review of the testing effect. *Psychological Bulletin,**140*(6), 1432–1463. 10.1037/a0037559 25150680 10.1037/a0037559

[CR95] Seabrooke, T., Hollins, T. J., Kent, C., Wills, A. J., & Mitchell, C. J. (2019a). Learning from failure: Errorful generation improves memory for items, not associations. *Journal of Memory and Language,**104*, 70–82. 10.1016/j.jml.2018.10.001

[CR96] Seabrooke, T., Mitchell, C. J., & Hollins, T. J. (2021a). Pretesting boosts item but not source memory. *Memory,**29*(9), 1245–1253. 10.1080/09658211.2021.1977328 34534032 10.1080/09658211.2021.1977328

[CR97] Seabrooke, T., Mitchell, C. J., Wills, A. J., & Hollins, T. J. (2021b). Pretesting boosts recognition, but not cued recall, of targets from unrelated word pairs. *Psychonomic Bulletin & Review*. 10.3758/s13423-020-01810-y 10.3758/s13423-020-01810-y 32959192

[CR98] Seabrooke, T., Mitchell, C. J., Wills, A. J., Inkster, A. B., & Hollins, T. J. (2022). The benefits of impossible tests: Assessing the role of error-correction in the pretesting effect. *Memory & Cognition,**50*(2), 296. 10.3758/S13421-021-01218-6 34363196 10.3758/s13421-021-01218-6 PMC8821051

[CR99] Seabrooke, T., Mitchell, C. J., Wills, A. J., Waters, J. L., & Hollins, T. J. (2019b). Selective effects of errorful generation on recognition memory: The role of motivation and surprise. *Memory,**27*(9), 1250–1262. 10.1080/09658211.2019.1647247 31369344 10.1080/09658211.2019.1647247

[CR100] Skinner, B. F. (1953). *Science and human behavior*. Simon and Schuster.

[CR101] Skinner, B. F. (1958). Teaching machines: From the experimental study of learning come devices which arrange optimal conditions for self-instruction. *Science,**128*(3330), 969–977. 10.1126/science.128.3330.969 13592277 10.1126/science.128.3330.969

[CR102] Slamecka, N. J., & Graf, P. (1978). The generation effect: Delineation of a phenomenon. Journal of Experimental Psychology: Human Learning & Memory, 4(6), 592–604. 10. 1037/0278–7393.4.6.592

[CR103] Stevenson, H. W., & Stigler, J. W. (1994). *The learning gap: Why our schools are failing and what we can learn from Japanese and Chinese education*. Simon & Schuster.

[CR104] Tanaka, S., Miyatani, M., & Iwaki, N. (2019). Response format, not semantic activation, influences the failed retrieval effect. *Frontiers in Psychology,**10*, 1–13. 10.3389/fpsyg.2019.00599 31019476 10.3389/fpsyg.2019.00599 PMC6459060

[CR105] Toftness, A. R., Carpenter, S. K., Lauber, S., & Mickes, L. (2018). The limited effects of prequestions on learning from authentic lecture videos. *Journal of Applied Research in Memory and Cognition,**7*(3), 370–378. 10.1016/j.jarmac.2018.06.003

[CR106] Tse, C. S., Balota, D. A., & Roediger, H. L., 3rd. (2010). The benefits and costs of repeated testing on the learning of face-name pairs in healthy older adults. *Psychology and Aging,**25*(4), 833–845. 10.1037/a0019933 20718541 10.1037/a0019933 PMC2990807

[CR107] Tulis, M. (2013). Error management behavior in classrooms: Teachers’ responses to student mistakes. *Teaching and Teacher Education,**33*, 56–68. 10.1016/j.tate.2013.02.003

[CR108] Tulis, M., Steuer, G., & Dresel, M. (2017). Positive beliefs about errors as an important element of adaptive individual dealing with errors during academic learning. *Educational Psychology,**38*(2), 139–158. 10.1080/01443410.2017.1384536

[CR109] Van den Broek, G., Takashima, A., Wiklund-Hornqvist, C., Wirebring, L. K., Segers, E., Verhoeven, L., et al. (2016). Neurocognitive mechanisms of the “testing effect”: A review. *Trends in Neuroscience and Education,**5*(2), 52–66. 10.1016/j.tine.2016.05.001

[CR110] van Gog, T., & Sweller, J. (2015). Not new, but nearly forgotten: The testing effect decreases or even disappears as the complexity of learning materials increases. *Educational Psychology Review,**27*(2), 247–264. 10.1007/s10648-015-9310-x

[CR111] Vaughn, K. E., & Rawson, K. A. (2012). When is guessing incorrectly better than studying for enhancing memory? *Psychonomic Bulletin & Review,**19*(5), 899–905. 10.3758/s13423-012-0276-0 22688538 10.3758/s13423-012-0276-0

[CR112] Vaughn, K. E., & Rawson, K. A. (2011). Diagnosing criterion-level effects on memory: What aspects of memory are enhanced by repeated retrieval? *Psychological Science,**22*(9), 1127–1131. 10.1177/0956797611417724 21813798 10.1177/0956797611417724

[CR113] Verhaeghen, P. (2016). Age-Related Slowing in Response Times, Causes and Consequences. In N. Pachana (Ed.), *Encyclopedia of Geropsychology*. Springer. 10.1007/978-981-287-080-3_211-2

[CR114] Yang, C., Potts, R., & Shanks, D. R. (2017). Metacognitive unawareness of the errorful generation benefit and its effects on self-regulated learning. *Journal of Experimental Psychology: Learning, Memory, and Cognition,**43*(7), 1073–1092. 10.1037/xlm0000363 28114778 10.1037/xlm0000363

[CR115] Wahlheim, C. N., & Jacoby, L. L. (2013). Remembering change: The critical role of recursive remindings in proactive effects of memory. *Memory & Cognition,**41*(1), 1–15. 10.3758/s13421-012-0246-9 22918874 10.3758/s13421-012-0246-9

[CR116] Wechsler, D. (2012). WAIS-IV. Escala de inteligencia de Wechsler para adultos-IV. Manual técnico y de interpretación. Pearson Educación.

[CR117] Wong, S. S. H., & Lim, S. W. H. (2019). Prevention–permission–promotion: A review of approaches to errors in learning. *Educational Psychologist,**54*(1), 1–19. 10.1080/00461520.2018.1501693

[CR118] Wong, S. S. H., & Lim, S. W. H. (2022). The derring effect: Deliberate errors enhance learning. *Journal of Experimental Psychology: General,**151*(1), 25–40. 10.1037/xge0001072 34242048 10.1037/xge0001072

[CR119] Zawadzka, K., & Hanczakowski, M. (2019). Two routes to memory benefits of guessing. *Journal of Experimental Psychology. Learning, Memory, and Cognition,**45*(10), 1748–1760. 10.1037/xlm0000676 30570326 10.1037/xlm0000676

[CR120] Zhao, B. (2011). Learning from errors: The role of context, emotion, and personality. *Journal of Organizational Behavior,**32*(3), 435–463. 10.1002/job.696

